# WorldwIde SurvEy on Clinical and Anatomical Factors Driving the Choice of Transcatheter Aortic Valve pRostheses

**DOI:** 10.3389/fcvm.2020.00038

**Published:** 2020-03-20

**Authors:** Luigi Biasco, Enrico Cerrato, Gregorio Tersalvi, Giovanni Pedrazzini, Ben Wilkins, Francesco Faletra, Enrico Ferrari, Stefanos Demertzis, Gaetano Senatore, Angelo Di Leo, Ferdinando Varbella, Ole De Backer, Luis Nombela Franco

**Affiliations:** ^1^Department of Biomedical Sciences, Università della Svizzera Italiana, Lugano, Switzerland; ^2^Division of Cardiology, Azienda Sanitaria Locale TO 4, Ciriè, Italy; ^3^Interventional Cardiology Unit, San Luigi Gonzaga University Hospital, Orbassano and Rivoli Infermi Hospital, Rivoli, Italy; ^4^Division of Cardiology, Cardiocentro Ticino, Lugano, Switzerland; ^5^Division of Cardiology, Rigshospitalet, Copenhagen, Denmark; ^6^Division of Cardiac Surgery, Cardiocentro Ticino, Lugano, Switzerland; ^7^Division of Cardiac Surgery, Cardiocentro Ticino, Lugano, Switzerland

**Keywords:** aortic stenosis, TAVI, TAVR, percutaneous aortic valve replacement, access, aortic annulus, survey

## Abstract

**Background:** Following the success of the first human transcatheter aortic valve replacement (TAVR) in 2002, multiple transcatheter heart valves (THVs) have become available. However, guidelines or expert consensus on how to optimize THV choice according to patients' anatomical and clinical characteristics is missing. This survey-based study aimed to identify patient-specific characteristics deemed important in the choice of THV type.

**Methods and results:** A web-based survey including 39 questions was completed by 71 experienced TAVR operators from 23 countries with a median TAVR volume of 88 procedures in the year prior to survey completion (IQR 61-180). The survey covered five topics: access, aortic annulus/leaflets, aortic root, left ventricular function and clinical characteristics. Factors with the most impact on THV choice were reported to be a calcified sinotubular junction, valve-in-valve procedure, annular dimension >575 mm^2^, femoral diameter ≤ 5.0 mm, low coronary ostia, calcification at the annular level and/or protruding into the left ventricular outflow tract, and need for post TAVR PCI. Also, in case of off-label use of THVs to treat bicuspid aortic valve disease and isolated aortic regurgitation, the choice of THV type was reported to be important.

**Conclusions:** This survey-based study identifies key patient characteristics that impact THV selection. As such, we present a guide, based on current practice, of which THV types are best suited to these different patient-specific characteristics. A patient-tailored THV choice is likely to optimize TAVR outcomes.

## Introduction

Since the first percutaneous transcatheter aortic valve replacement (TAVR) performed in 2002 (1), a range of percutaneous aortic prostheses have been released by numerous manufacturers. Initial devices were designed, trial-tested and released for use in patients with severe symptomatic aortic valve stenosis and contraindication to surgery (2). These early trials have informed current TAVR exclusion/inclusion criteria in real-world settings. With progress in the field of TAVR, extension of the indications to lower risk patients has widened the treatable population and its clinical and anatomical landscape (3).

While producing TAVR systems for a wide range of patients, manufacturers have developed significantly different prostheses in terms of valve design, release technology, and delivery systems (4). In keeping with this, various patient characteristics have influence over which device becomes more individually favorable. Vascular access, tortuosity, potential complications, aortic anatomy, calcifications, conduction disturbances and comorbidities must be considered in the complex pre-procedural planning of TAVR and valve choice.

Criteria on a precise matching between different prostheses and patients' characteristics are not well defined. Nonetheless, TAVR operators are aware that interaction between the patient and device can have a crucial influence on procedural success, valve durability, and potentially on long term outcomes (5).

Recently, a European survey generically defined “patient's specific characteristics” as the main criterion guiding the selection of a specific prosthesis (6). In addition, the European guidelines (ESC/EACTS) on valvular heart diseases recognized an initial set of general, anatomical, and technical aspects that should be considered by the Heart Team for the decision between surgical aortic valve replacement and TAVR in patients at increased surgical risk (2).

We therefore designed the WISE-TAVR survey, to clearly identify clinical and anatomical characteristics deemed relevant in patient-prosthesis matching by a collective of expert TAVR operators worldwide. Our aim was to investigate and describe the clinical and anatomical aspects having an impact on the operator's choice for a particular transcatheter heart valve (THV) and to provide a guide for a modern patient-tailored THV therapy.

## Materials and Methods

### Survey Design

The survey was designed by a team of physicians with experience in TAVR (LB, EC, OD, LN). The survey engine was built under supervision of one of the investigators (EC) using a dedicated online platform hosted on a collaborative research website (www.cardiogroup.org) and included a total of 39 questions with single, multiple choice and open-ended answering options. The full survey was designed to address five major domains related to TAVR: vascular access, aortic annulus/aortic leaflets, aortic root, left ventricular, and clinical characteristics.

In order to minimize possible misunderstandings in interpreting questions, each scenario was correlated by a clear definition and a descriptive image. Definitions of anatomical characteristics have been derived from current literature or inclusion/exclusion criteria of randomized clinical trials or CE mark trials when available. In all other cases, a working definition was developed. All definitions are available within survey text (available as an online Supplementary Material).

For each clinical/anatomical scenario two questions were proposed. As a first TAVR operators were asked whether the proposed characteristics would impact on the choice of the prosthesis and a yes/no answer allowed. Then a second question investigated how would they grade the suitability of the most widely used THV according to the proposed scenario. According to the commercial availability of different THV at the time of survey's design five different platforms were investigated: Edwards Sapien XT/3, Medtronic CoreValve Evolut R, Boston Lotus, Boston Acurate Neo, and Abbot Portico. Responders could choose whether to answer or skip the proposed question.

Distribution of the survey was web- and mail-based aiming at covering different geographic areas. More than 200 operators with expertise on TAVR were contacted. Data acquisition was kept open for 3 months. All forms were electronically acquired, and data analyzed. The software allowed monitoring of results at all times. An ongoing monitoring for survey accrual and completion was then performed in order to avoid duplicate entry or missing data. Incomplete forms were defined as those with empty fields regarding questions 1–5 and with <80% of remaining answers provided. Participation was purely voluntary and unpaid, and all responses were confidential. This survey was investigator initiated. No support from industry was received for design, development of the online platform, data acquisition/analysis or writing. Ethical review process was not required for our study, since according to the Swiss law Cantonal ethics committee is only responsible for the examination and authorization of research projects conducted on human beings, thus not applying to our setting.

### Statistical Analysis

Categorical variables are expressed as percentages and continuous variables as mean (± standard deviation) or median (interquartile [IQR]: 25-75th percentile or range: minimum-maximum) according to variable distribution. All analyses were performed using SPSS 20 (IBM, Armonk, NY, USA).

Factors determining the choice of THV have been split into three categories: Low (<60% of responders agreed on their role in the choice of THV), intermediate (≥60 and <80% of responders), and definite impact (≥80% of responders agreed on their role in the choice of THV). When a clinical or anatomical characteristic was recognized having a definite impact on the choice of THV (>80%) but no consensus was reached on THV prosthesis selection (i.e., when reaching <80% of operator preferences), this was identified as an unmet need from current devices.

## Results

### Participants

A total of 89 questionnaires were returned. Of these, 18 were excluded for significant data incompleteness. 71 responses were analyzed including 52 European (73%), 6 North American (8%), 5 Central and South American (7%), and 8 Asia or Australia (11%). Most responders were interventional cardiologists (91%), while the remaining were cardiac surgeons (6%) or imaging specialists (3%).

Participating centers had a median experience of 500 total TAVR procedures (IQR: 293 to 825 procedures), with 88 (IQR 61–180), procedures in the year prior to survey completion. An invitation to tender for THV equipment was declared by 42% of responders while 36% report the presence of package deals in their center.

### Procedures

Eighty-four percent of responders reported a preference for local analgesia with conscious sedation. 64% offer alternative access TAVR either via trans-subclavian/axillary, transaortic, transcaval, or transapical approach. Only one device was available in 6% of centers while 37% reported to implant at least 2 different prostheses, 14% 3 devices, 27% 4 devices and 16% more than 4 devices. Almost half (49%) of clinicians reported CoreValve (Medtronic, Minneapolis, Minnesota) being their most-used valve, followed by SAPIEN XT or 3 (41%) (Edwards Lifesciences, Irvine, California), Portico (4%) (Abbott, Santa Clara, California), and Acurate Neo (3%) (Boston Scientific, Marlborough, Massachusetts).

### Anatomical Characteristics Guiding the Choice of TAVR

#### Access

**Figure 1:**
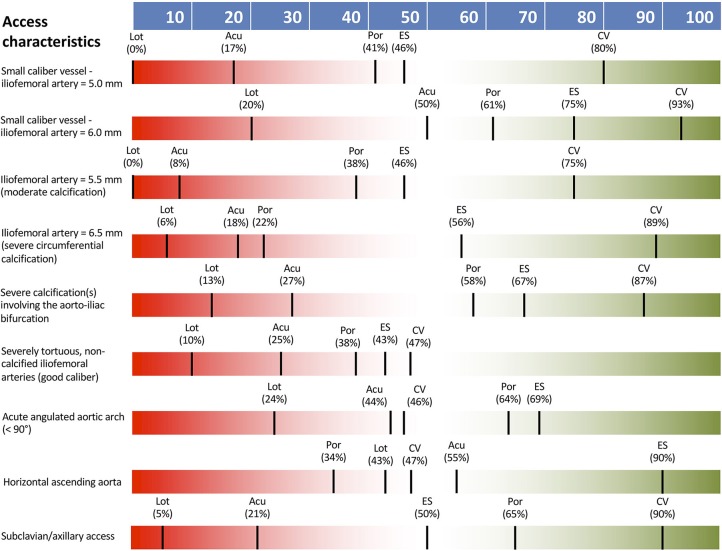
THV preferences according to access vessel characteristics. Acu, Boston Acurate Neo; CV, Medtronic CoreValve; ES, Edwards Sapien XT/3; Lot, Boston Lotus; Por, Abbott Portico.

THV type selection was definitely impacted (> 80% responder agreement) by the presence of femoral caliber of 5.0 mm without significant calcification or 5.5 mm with moderate calcification. The presence of other vascular access characteristics including: iliofemoral artery 6.0 mm, iliofemoral artery 6.5 mm with severe circumferential calcification, severe calcification of aortoiliac bifurcation, severely tortuous, non-calcified iliofemoral arteries, acute aortic arch angulation >90°, horizontal ascending aorta, and subclavian/axillary access had either low or intermediate impact only on THV type selection. None of the listed vascular access characteristics was considered as definitely contraindicating femoral access. THV preference according to access characteristics are shown in Figure 1, with a general preference for the CoreValve TVH in cases of challenging access.

#### Characteristics of the Aortic Annulus

Large annular area between 575 and 660 mm^2^ had a definite impact on THV type selection with a consensus for both CoreValve and Sapien platforms. Even larger anatomy (annulus area > 660 mm^2^) or calcification of the left ventricular outflow tract (LVOT) extending >5 mm into the lumen also had a definite impact on TVH type selection but no consensus on THV type was seen. A definite impact on THV type choice was seen in cases of severe annular calcification extending >5 mm into the lumen, with preference for CoreValve. Small annular area (<325 mm^2^) and severe annular calcification had an intermediate effect on THV selection. Ellipticity index >2.5 had a low impact on THV selection.

**Figure 2:**
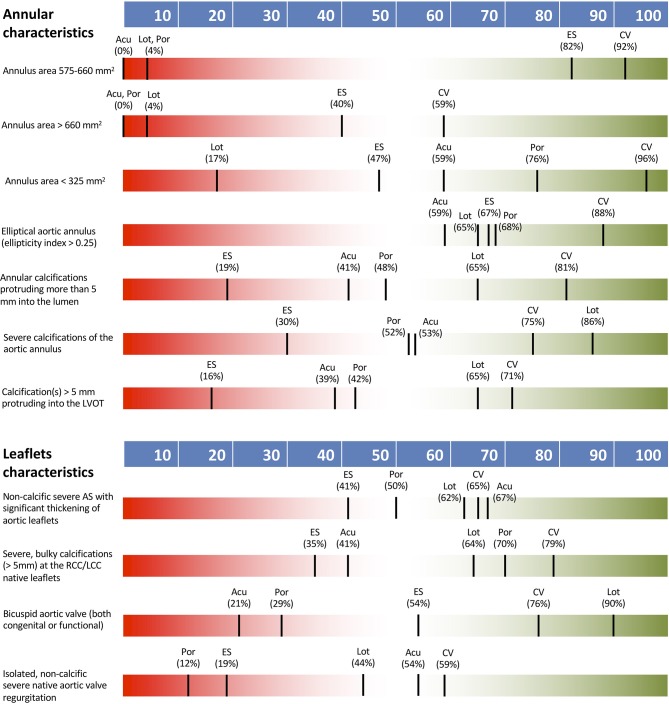
THV preferences according to annular and leaflet characteristics. Acu, Boston Acurate Neo; CV, Medtronic CoreValve; ES, Edwards Sapien XT/3; Lot, Boston Lotus; Por, Abbott Portico.

Figure 2 shows THV preferences according to annular and leaflet characteristics.

#### Characteristics of the Leaflets

Bicuspid aortic stenosis or isolated non-calcific, native valve, severe aortic regurgitation (AR) had a definite impact on THV type selection. In the presence of bicuspid anatomy consensus preference was for Lotus THV, whereas no consensus was noted on specific valve choice for aortic regurgitation. Significant non-calcific thickening of aortic leaflets or severe bulky calcifications of the coronary cusps were not recognized as determinants on the choice of THV.

#### Aortic Root Characteristics

Aortic root characteristics were generally seen as important to guiding THV selection, with low coronary take-off, small sinus of Valsalva, calcified and small diameter sinotubular junction and valve-in-valve TAVR procedure all having a definite impact. For valve-in-valve cases, the CoreValve system was identified as most suitable, with no other consensus being reached for TVH selection. A dilated ascending aorta had only low impact on THV type selection.

#### Left Ventricular and Clinical Characteristics

Left ventricular function, conduction status and various clinical characteristics were not seen as key determinants of TVH type selection with no definite impact on THV type noted. The only factor with a definite impact was need for PCI following TAVR (80% of responders) where 98% of operators identified the Sapien valve as a suitable choice in this scenario. LVEF <35%, RBBB or LBBB, first-degree AVB, younger patient age (65–70 years) and severe LV hypertrophy with small cavity had either low or intermediate impact on TVH type selection only.

**Figure 3:**
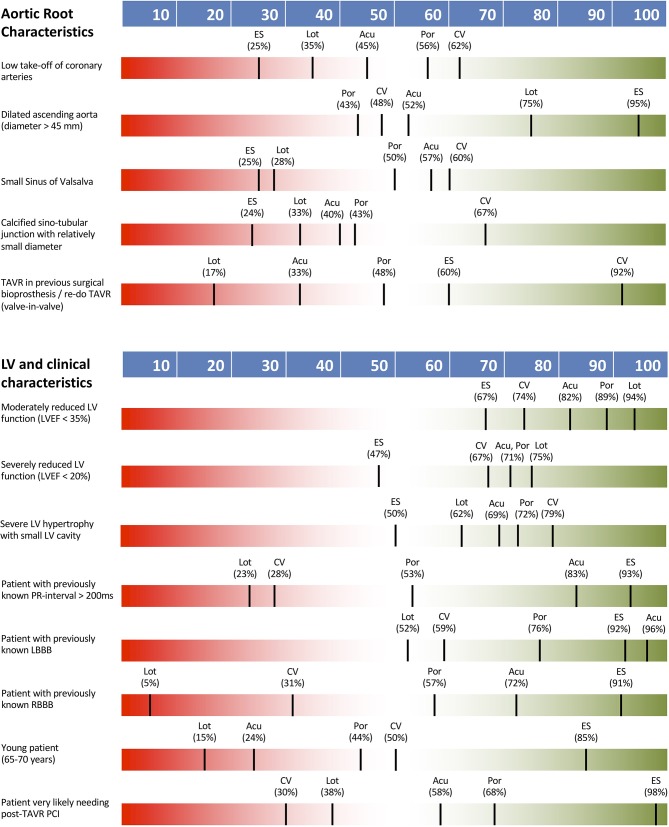
THV preferences according to aortic root and clinical characteristics. Acu, Boston Acurate Neo; CV, Medtronic CoreValve; ES, Edwards Sapien XT/3; Lot, Boston Lotus; Por, Abbott Portico.

Figure 3 shows THV preferences according to aortic root and clinical characteristics. Table 1 summarizes survey's findings by reporting the percentage of responders declaring an impact of the proposed clinical/anatomical scenario on the choice of a peculiar THV.

**Table 1:** Summary of survey's findings: percent of responders reporting impact on the choice of THV for each clinical/anatomical characteristic.

	**Impact on choice of THV (%)**	**Impact on choice of access (%)**
**Definite impact (>80%)**		
Calcified sino-tubular junction with small diameter	94	
Iliofemoral artery = 5.0 mm	90	42
Large-sized aortic annulus (area 575–660 mm^2^)	90	
TAVR in previous surgical bioprosthesis (valve-in-valve)	90	
Severe calcification(s) > 5 mm protruding into the LVOT	89	
Low take-off of coronary arteries	89	
Iliofemoral artery 5.5 mm (moderate calcifications)	85	34
Large-sized aortic annulus (area > 660 mm^2^)	85	
Small sinus of Valsalva	84	
Aortic Regurgitation	83	
Bicuspid Aortic Valve	83	
Severe annular calcifications (>5 mm)	81	
Needing PCI	80	
**Intermediate impact (60–80%)**		
Small-sized aortic annulus (area < 325 mm^2^)	78	
Severe calcification(s) of the aortic annulus	76	
Subclavian/axillary access	73	
Horizontal ascending aorta	72	10
Iliofemoral artery = 6.5 mm circumferential calcifications	71	31
Acute angulated aortic arch	71	22
LVEF < 20%	67	
Young patients	65	
Severely tortuous, non-calcified iliofemoral arteries	64	39
Severe calcification(s) involving the aorto-iliac bifurcation	62	23
Non-calcific severe AS-thick leaflets	62	
RBBB	61	
Severe calcifications (>5 mm) at the RCC/LCC leaflets	61	
**Low impact (<60%)**		
Patient with previously known PR-interval > 200 ms	51	
Hypertrofic LV	49	
Ilio-femoral arteries = 6 mm, no calcification	47	6
Elliptical aortic annulus	47	
Dilated ascending aorta	41	
LVEF < 35%	32	
LBBB	28	

## Discussion

During early experience with TAVR, anatomical criteria from initial randomized clinical trials (6–11) served as a reference for case selection. Now, extension of TAVR into wider patient groups (e.g., intermediate, low or extremely high-risk surgical patients) or untrialled clinical situations (e.g., aortic regurgitation or valve in valve procedures) has created a gap between the original trial guidelines and current accepted practice (Supplementary File 1). For these reasons, application of previous TAVR trial inclusion/exclusion criteria in a real-life scenario is challenging if not outdated.

The institutional norm for using multiple parallel THV options points to the various strengths and weakness of each system and confirms that no system has yet achieved a “one size fits all” status. This highlights the importance of optimized patient-THV matching, an area of TAVR practice that is currently lacking in guidelines or consensus. Data derived from WISE-TAVR allows a snapshot of current practice according to patient specific characteristics in high volume centers and by experienced operators. It also identifies aspects of current TAVR practice that have unmet needs and provides clues for the technical development of next generation prostheses.

We have identified a specific set of clinical and anatomical characteristics which currently impact the choice of THV for patients. In particular, small access vessel caliber < 5.0 mm non-calcified or < 5.5 mm with moderate vessel calcification, large annulus dimension > 575 mm^2^, small sinus of Valsalva, low take off of coronary arteries, annular calcifications > 5 mm protruding into the lumen at the annulus or LVOT, a small, calcific sinotubular junction, valve in valve procedures, bicuspid aortic valve, aortic regurgitation and need for PCI have the most effect on the current choice of THV type, with ≥ 80% of TAVR operators agreeing on their impact.

In addition, we identify that several patient characteristics considered as exclusion criteria from most controlled trials or potentially hampering procedural success of TAVR (e.g., horizontal aorta, tortuous accesses, severe LV impairment, dilated ascending aorta) are not perceived as factors either to deny TAVR or to strongly impact the choice of a particular THV.

There is agreement that within the range of TAVR devices, some are more suited to specific clinical and patient conditions.

While the cilindric, short frame Edwards Sapien valve is almost universally preferred in AS patients likely to undergo PCI after TAVR, other platforms extending into the proximal portion of ascending aorta such as Corevalve were reported as unsuitable/not preferred.

Due to intrinsic platforms limitations correlated with the currently commercially available sizes, Lotus and Acurate Neo and Portico were deemed unsuitable in large anatomies. Conversely both CoreValve and Sapien platforms, due to valve design and peculiar implant techniques, were considered appropriate choices in this setting. *Vice versa*, when facing small annular dimensions, self-expandable prostheses with dedicated small platforms such as the Portico and CoreValve were preferred over other devices. Smaller and flexible delivery systems such as the Medtronic Enveo R or the Portico delivery system were preferred in complex femoral accesses.

Of interest, and potentially based on initial evidences (12), presence of preprocedural conduction disturbances, and in particular right bundle branch block or first degree AV block resulted one of the main criterion the support the choice of Acurate Neo, due to correlated low incidence of pacemaker implant.

However, when facing complex anatomical challenges, opinions frequently diverge on as to which device is most appropriate. In this situation, local device availability, technical skills, personal preferences, and cost are likely to have an impact on THV selection. This is consistent with the WRITTEN survey, performed to obtain a global view of current practice related to TAVR which emphasized the presence of several areas of divergence in TAVR procedural details and patient's management between centers (13).

Several anatomical features still represent an unmet need for currently commercially available prostheses. Annular dimensions exceeding 660 mm^2^, low take-off coronary ostia, small sinus of Valsalva, small, calcified sinotubular junction, and isolated aortic regurgitation, while being recognized as factors impacting on THV choice, were not well matched to any currently available device. These characteristics represent challenges to be considered in the technical development of future THV, allowing more consistent, successful treatment of these challenging patient groups.

## Limitations

Several limitations of the present survey should be acknowledged. Firstly, despite the attempt to achieve a wide response, only a third of invited specialists completed the survey. Because of market distribution and global TAVR practice, responders were mainly CoreValve or Sapien users, while the remaining prostheses were used less frequently, potentially impacting on the reported preferences. Due to the recent implementation of newer THV generations after completion of this survey, we are not able to provide data for all currently commercially available devices (e.g., CoreValve PRO).

In addition results were not weighted against operator/center volumes.

## Conclusion

In this study a set of characteristics was identified as having definite impact on the suitability of a patient for TAVR and the type of THV selected. In addition, several of these characteristics have clear consensus for which specific THV is most appropriate, representing an initial guide for current international practice. Other anatomical conditions were identified as definite factors impacting on THV choice but were not well matched to any specific THV—these represent unmet needs to be considered in the technical development of future THV.

## Data Availability Statement

The datasets generated for this study are available on request to the corresponding author.

## Author Contributions

LB, OD, LN, and EC developed the survey, analyzed the data, and drafted the manuscript. GT, GP, BW, FF, EF, SD, GS, AD, and FV critically reviewed the manuscript.

### Conflict of Interest

The authors declare that the research was conducted in the absence of any commercial or financial relationships that could be construed as a potential conflict of interest.

## Supplementary Material

Supplementary File 1 Survey text.

Supplementary File 2 Summary of exclusion criteria used in randomized clinical trials/CE mark trials according to different THV devices.
