# Supplementary material for: WorldwIde SurvEy on Clinical and Anatomical Factors Driving the Choice of Transcatheter Aortic Valve pRostheses
Source: Front Cardiovasc Med. 2020 Mar 20;7:38. doi: 10.3389/fcvm.2020.00038 (PMC7098951; doi:10.3389/fcvm.2020.00038)
Supplement: Supplementary File 1 — Survey text. [file Data_Sheet_1.PDF]

CMS Survey

Project

Provided by www.mydataservice.org
International collaborative group for research projects

SURVEY

Just a sample of possible questions

Question 1

What is your specialty?

- ○ Interventional cardiologist
- ○ Cardiac surgeon
- ○ Fellow of interventional cardiology
- ○ Fellow of cardiac surgery
- ○ Imaging specialist
- ○ Other

What is your country of clinical practice? Italy

Question 2

When was the first TAVI procedure performed at your center?
How many TAVI procedures have been performed at your center to date?
How many TAVI procedures were performed at your center last year?
How many TAVI procedures have you performed last year?
What is the portion of TAVI procedures performed under general anaesthesia?

Question 3

What types of transcatheter heart valves (THVs) are available at your site? Please indicate the most frequently used THV in 2016-2017 with "1", the second most used THV with "2", etc.

o Sapien 3 (Edwards Lifesciences)
o Corevalve Evolut R (Medtronic)
o Lotus Valve System (Boston Scientific)
o Symetis Acurate neo (Boston Scientific)
o Portico (Abbott)
o Direct Flow
o Engager (Medtronic)
o Jena Valve (JenaValve Technology)
o Heart Leaflet Technology
o Other

Question 4

Who is involved at your site in the choice of the type of THV implanted?

- ○ First operator only
- ○ Team of operators
- ○ Heart Team (including at least interventional cardiologists and imaging specialist)

Is there an invitation to tender for THV equipment at your center? ○ Yes ○ No
Does your center have any 'package deals' with some of the THV vendors? ○ Yes ○ No
Are you involved in proctoring activities? ○ Yes ○ No, if yes please specify the manufacturer

Question 5

Which TAVI access routes are used at your center? Please indicate the estimated use of each access route in % at your center within the last year.

- ○ Transfemoral ____ %
- ○ Transsubclavian/transaxillary ____ %
- ○ Direct aortic ____ %
- ○ Transapical ____ %
- ○ Transcaval ____ %

Question 6

Small caliber vessel - iliofemoral artery = 5.0 mm (absence of or only mild calcification)
- Would this anatomical finding let you choose an alternative, non-TF access route? - ○ Yes ○ No
- Would this anatomical finding impact your choice of THV? - ○ Yes ○ No
- How suitable would you grade the following THVs?

Sapien 3 (Edwards): -
Corevalve Evolut R (Medtronic): -
Lotus Valve (Boston Scientific): -
Symetis Acurate (Boston Scientific): -
Portico (Abbott): -

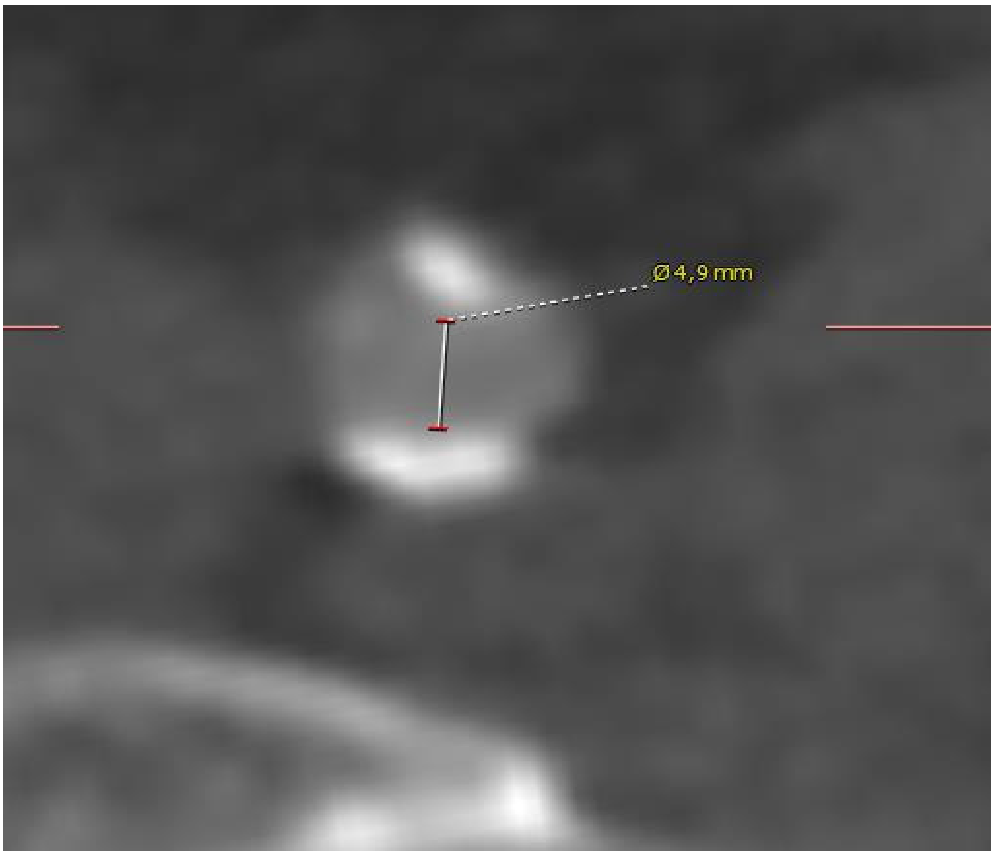

Question 7

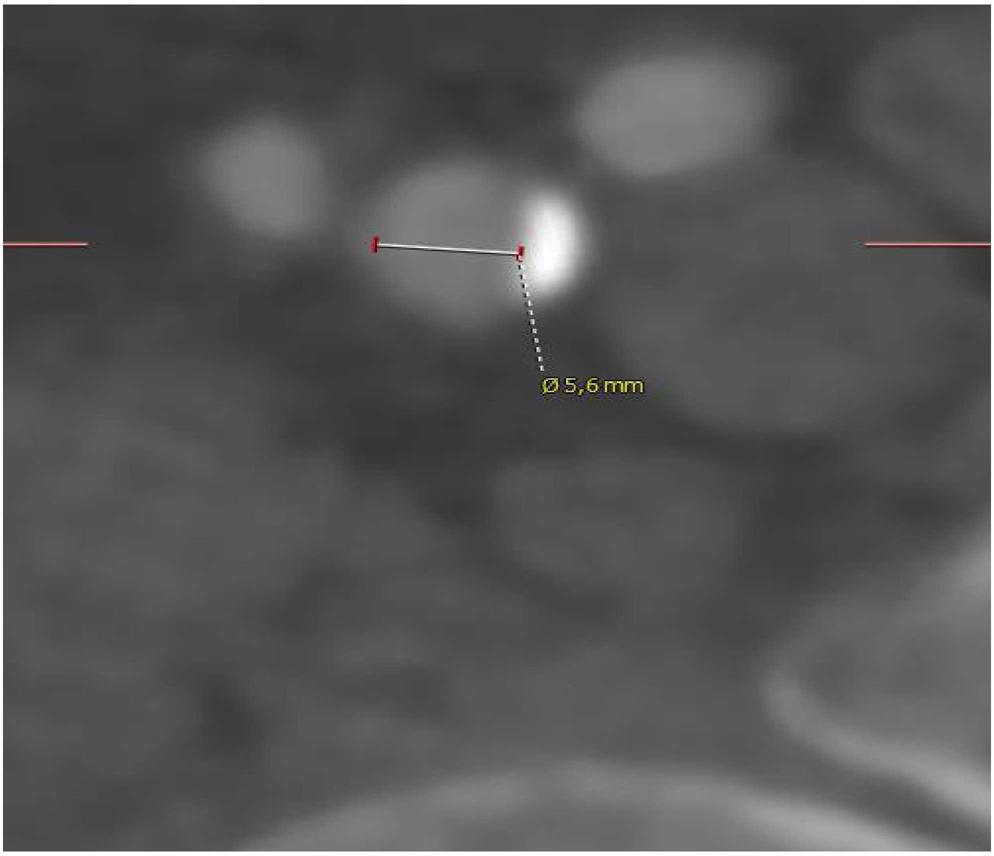

Question 8

Small caliber vessel - iliofemoral artery = 5.5 mm (moderate calcification)
- Would this anatomical finding let you choose an alternative, non-TF access route? - ○ Yes ○ No
- Would this anatomical finding impact your choice of THV? - ○ Yes ○ No
- How suitable would you grade the following THVs?

Sapien 3 (Edwards): -
Corevalve Evolut R (Medtronic): -
Lotus Valve (Boston Scientific): -
Symetis Acurate (Boston Scientific): -
Portico (Abbott): -

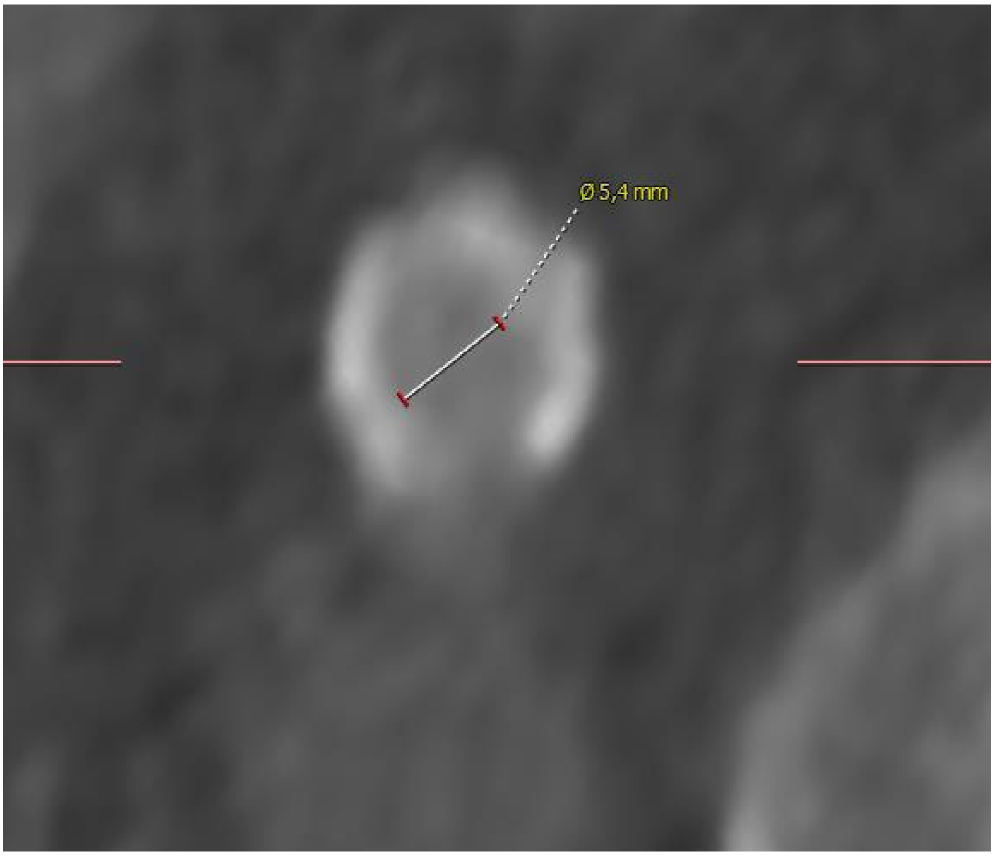

Question 9

Small caliber vessel - iliofemoral artery = 6.5 mm (severe circumferential calcification)
- Would this anatomical finding let you choose an alternative, non-TF access route? - ○ Yes ○ No
- Would this anatomical finding impact your choice of THV? - ○ Yes ○ No
- How suitable would you grade the following THVs?

Sapien 3 (Edwards): -
Corevalve Evolut R (Medtronic): -
Lotus Valve (Boston Scientific): -
Symetis Acurate (Boston Scientific): -
Portico (Abbott): -

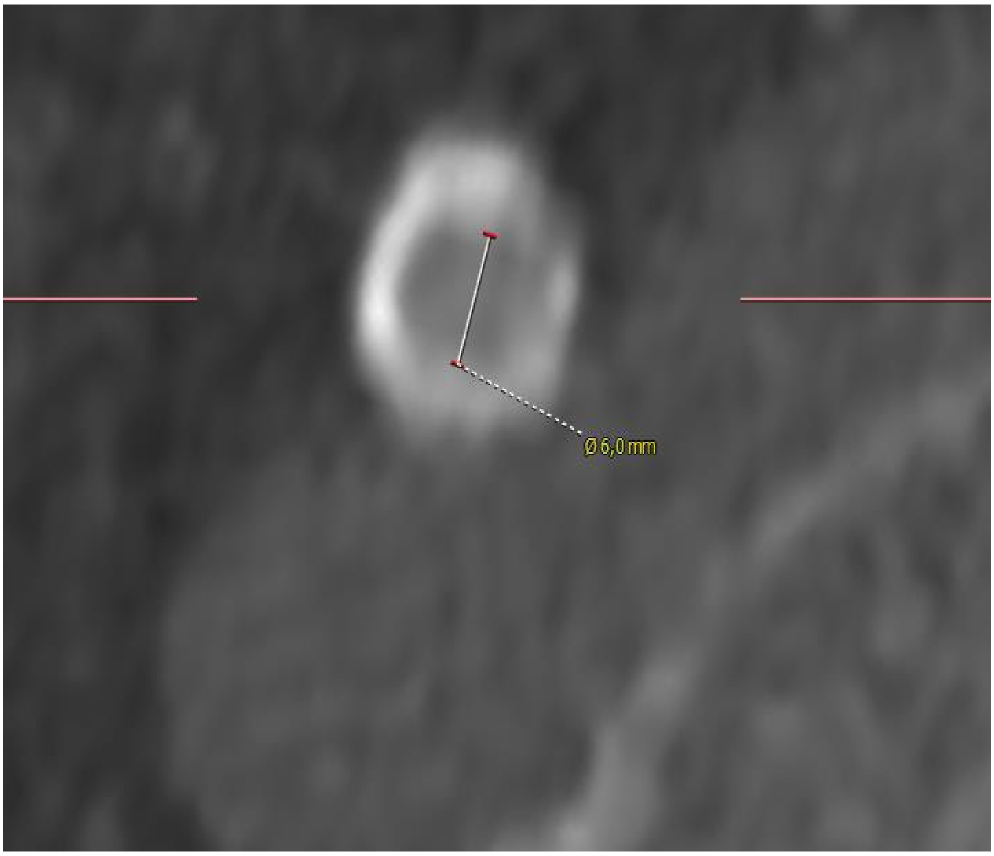

Question 10

Severe calcification(s) involving the aorto-iliac bifurcation
- Would this anatomical finding let you choose an alternative, non-TF access route? - ○ Yes ○ No
- Would this anatomical finding impact your choice of THV? - ○ Yes ○ No
- How suitable would you grade the following THVs?

Sapien 3 (Edwards): -
Corevalve Evolut R (Medtronic): -
Lotus Valve (Boston Scientific): -
Symetis Acurate (Boston Scientific): -
Portico (Abbott): -

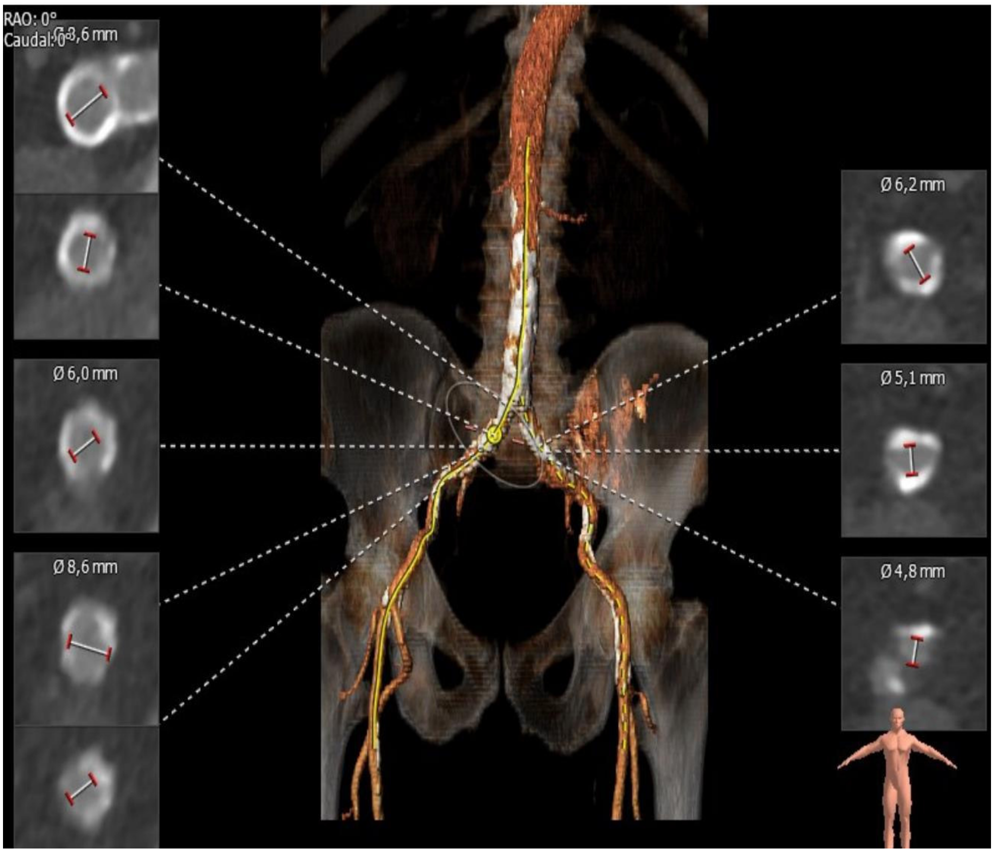

Question 11

Severely tortuous, non-calcified iliofemoral arteries (good caliber)
- Would this anatomical finding let you choose an alternative, non-TF access route? - ○ Yes ○ No
- Would this anatomical finding impact your choice of THV? - ○ Yes ○ No
- How suitable would you grade the following THVs?

Sapien 3 (Edwards): -
Corevalve Evolut R (Medtronic): -
Lotus Valve (Boston Scientific): -
Symetis Acurate (Boston Scientific): -
Portico (Abbott): -

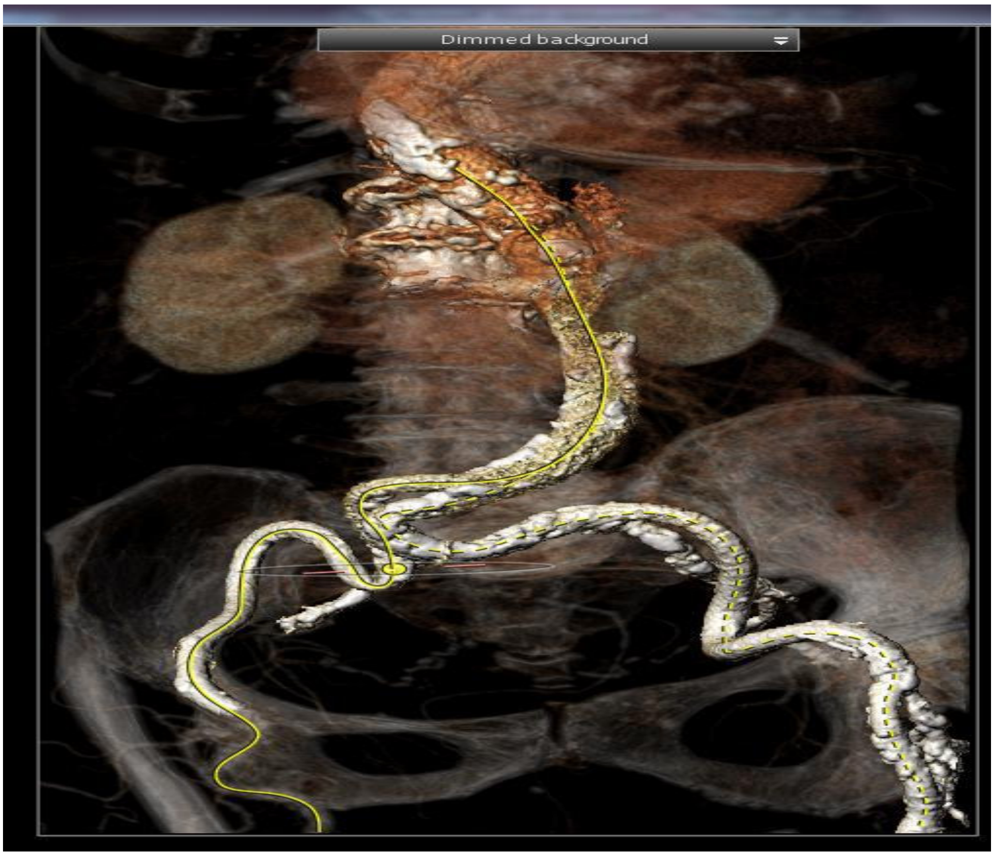

Question 12

Acute angulated aortic arch (<90 ; gothic aortic arch)

- Would this anatomical finding let you choose an alternative, non-TF access route? - ○ Yes ○ No

- Would this anatomical finding impact your choice of THV? - ○ Yes ○ No

- How suitable would you grade the following THVs?

Sapien 3 (Edwards): -

Corevalve Evolut R (Medtronic): -

Lotus Valve (Boston Scientific): -

Symetis Acurate (Boston Scientific): -

Portico (Abbott): -

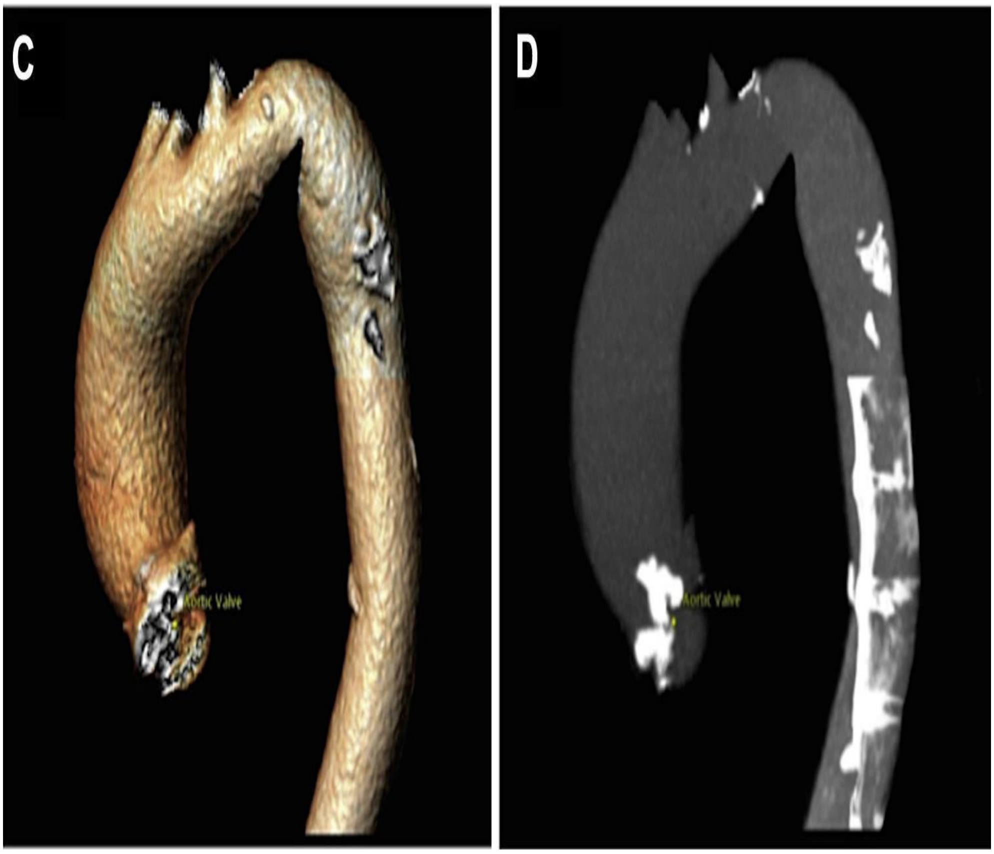

Question 13

Horizontal ascending aorta (angle between aortic annulus and horizontal plane > 70)
- Would this anatomical finding let you choose an alternative, non-TF access route? - ○ Yes ○ No
- Would this anatomical finding impact your choice of THV? - ○ Yes ○ No
- How suitable would you grade the following THVs?

Sapien 3 (Edwards): -
Corevalve Evolut R (Medtronic): -
Lotus Valve (Boston Scientific): -
Symetis Acurate (Boston Scientific): -
Portico (Abbott): -

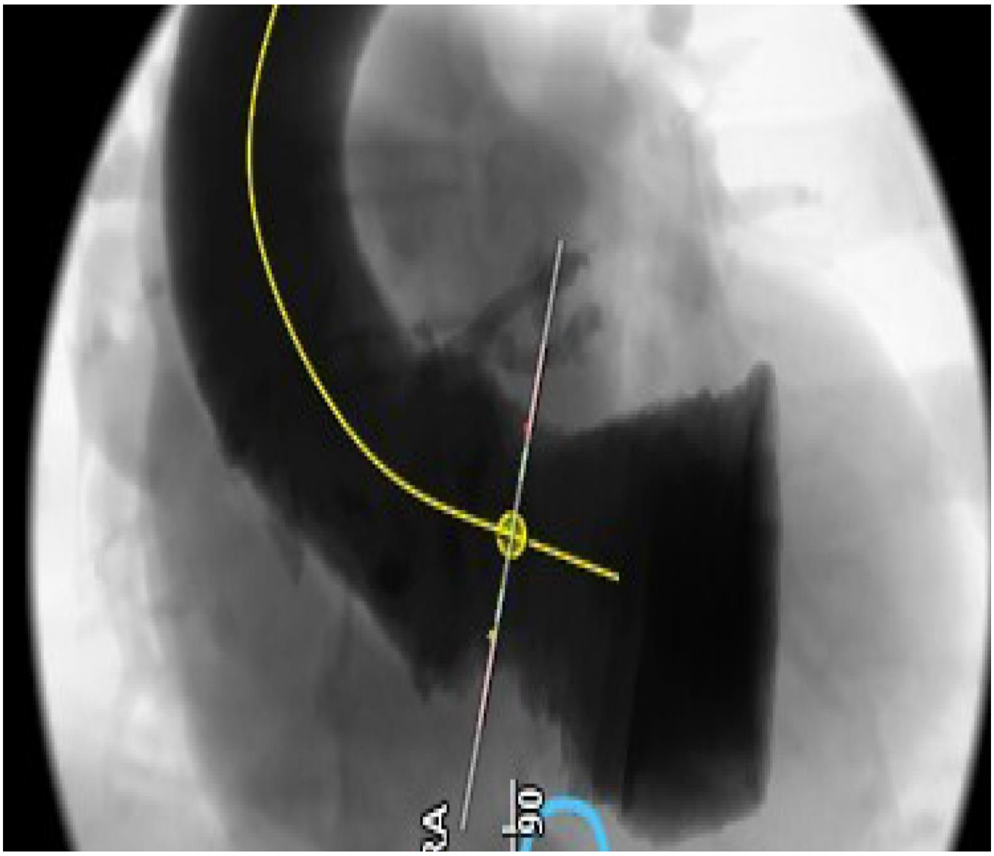

Question 14

Subclavian/axillary access
- Would this decision impact your choice of THV? - ○ Yes ○ No
- How suitable would you grade the following THVs?

Sapien 3 (Edwards): -
Corevalve Evolut R (Medtronic): -
Lotus Valve (Boston Scientific): -
Symetis Acurate (Boston Scientific): -
Portico (Abbott): -

Question 15

Large-sized aortic annulus (area 575-660 mm3, perimeter 85-90 mm, mean diameter 27-29)
- Would this anatomical finding impact your choice of THV? - ○ Yes ○ No
- How suitable would you grade the following THVs?

Sapien 3 (Edwards): -
Corevalve Evolut R (Medtronic): -
Lotus Valve (Boston Scientific): -
Symetis Acurate (Boston Scientific): -
Portico (Abbott): -

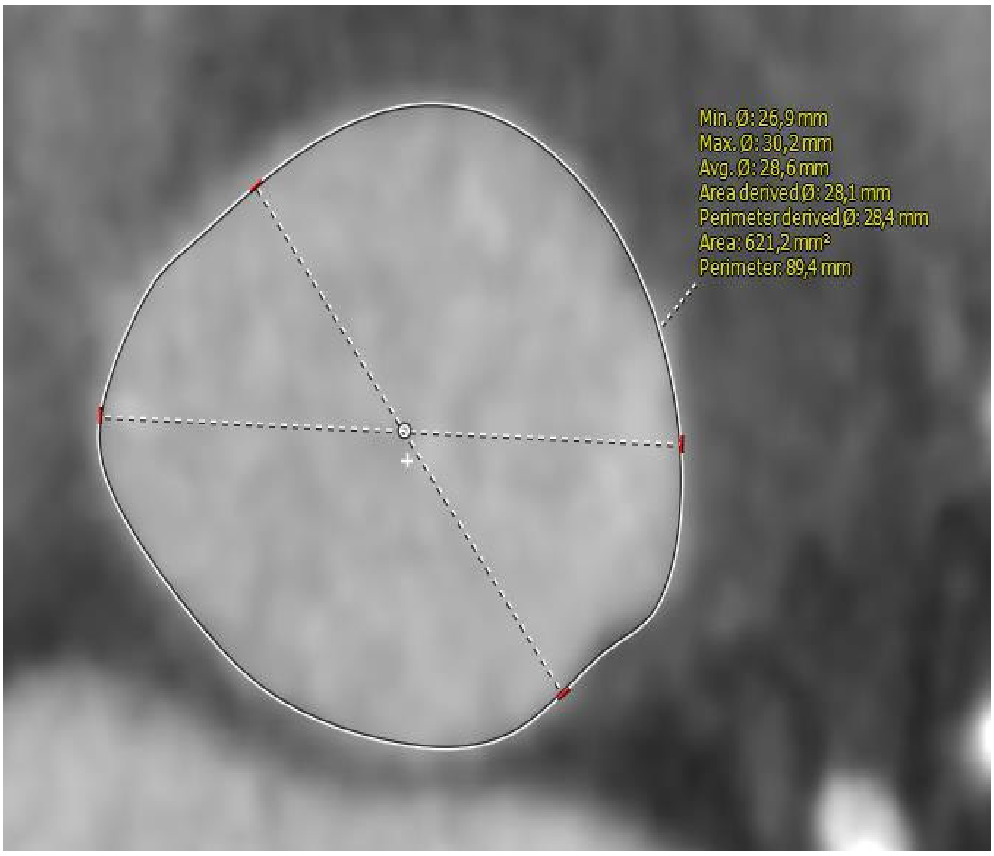

Question 16

Large-sized aortic annulus (area > 660 mm3, perimeter > 90 mm, mean diameter > 29)
- Would this anatomical finding result in denying TAVI in case of tricuspid AS? - ○ Yes ○ No
- Would this anatomical finding result in denying TAVI in case of bicuspid AS? - ○ Yes ○ No
- Would this anatomical finding impact your choice of THV? - ○ Yes ○ No
- How suitable would you grade the following THVs?

Sapien 3 (Edwards): -
Corevalve Evolut R (Medtronic): -
Lotus Valve (Boston Scientific): -
Symetis Acurate (Boston Scientific): -
Portico (Abbott): -

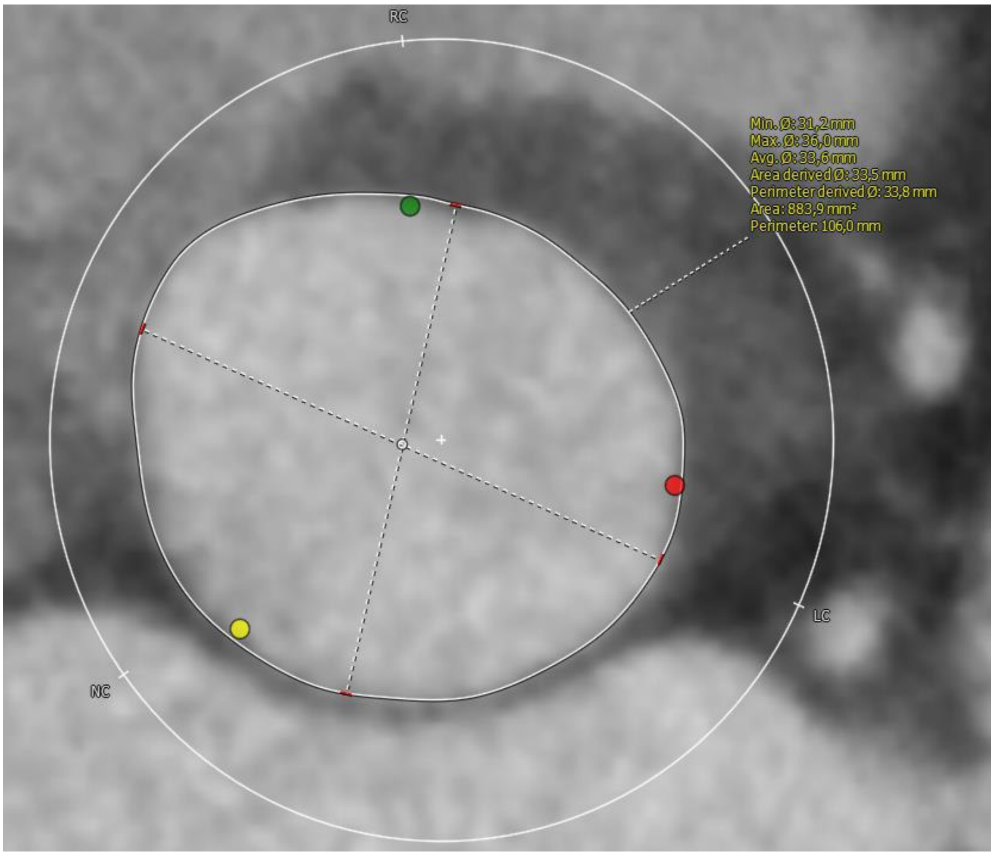

Question 17

Small-sized aortic annulus (area < 325 mm3, perimeter < 64 mm, mean diameter < 20)
- Would this anatomical finding impact your choice of THV? - ○Yes ○No
- How suitable would you grade the following THVs?

Sapien 3 (Edwards): -
Corevalve Evolut R (Medtronic): -
Lotus Valve (Boston Scientific): -
Symetis Acurate (Boston Scientific): -
Portico (Abbott): -

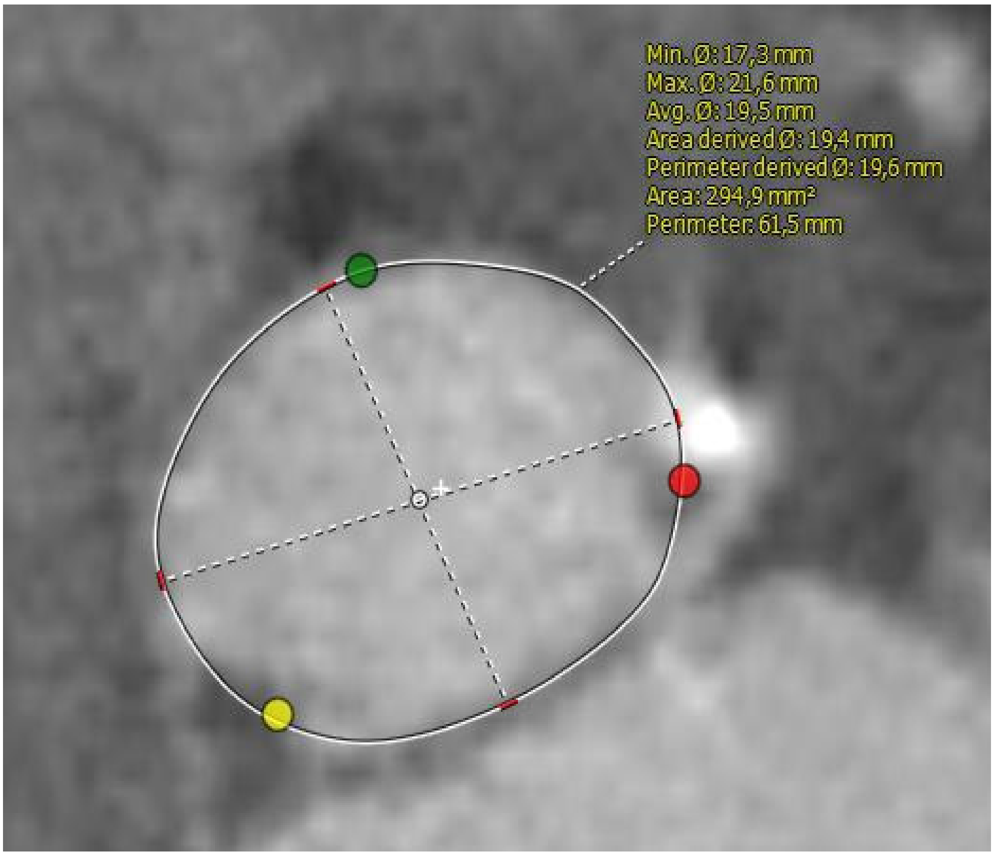

Question 18

Elliptical aortic annulus (ellipticity index > 0.25)

- Would this anatomical finding impact your choice of THV? - ○Yes ○No

- How suitable would you grade the following THVs?

Sapien 3 (Edwards): -

Corevalve Evolut R (Medtronic): -

Lotus Valve (Boston Scientific): -

Symetis Acurate (Boston Scientific): -

Portico (Abbott): -

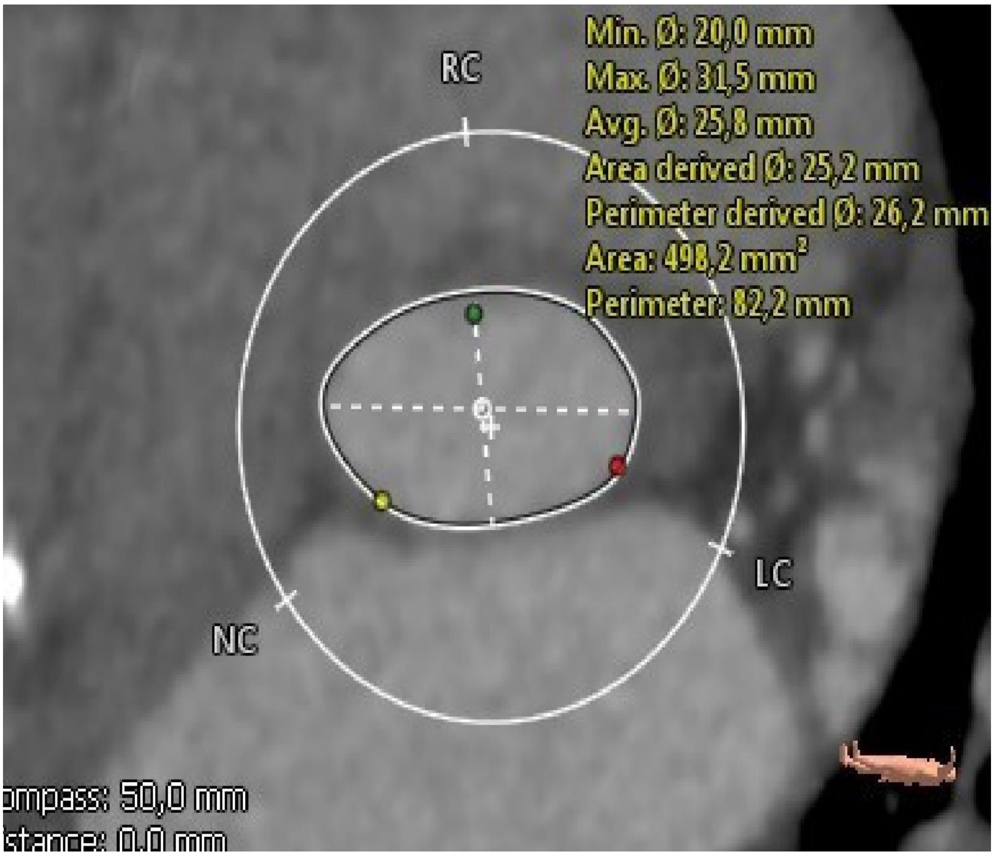

Question 19

Severe annular calcification(s) protruding more than 5 mm into the lumen
- Would this anatomical finding impact your choice of THV? - ○ Yes ○ No
- How suitable would you grade the following THVs?

Sapien 3 (Edwards): -
Corevalve Evolut R (Medtronic): -
Lotus Valve (Boston Scientific): -
Symetis Acurate (Boston Scientific): -
Portico (Abbott): -

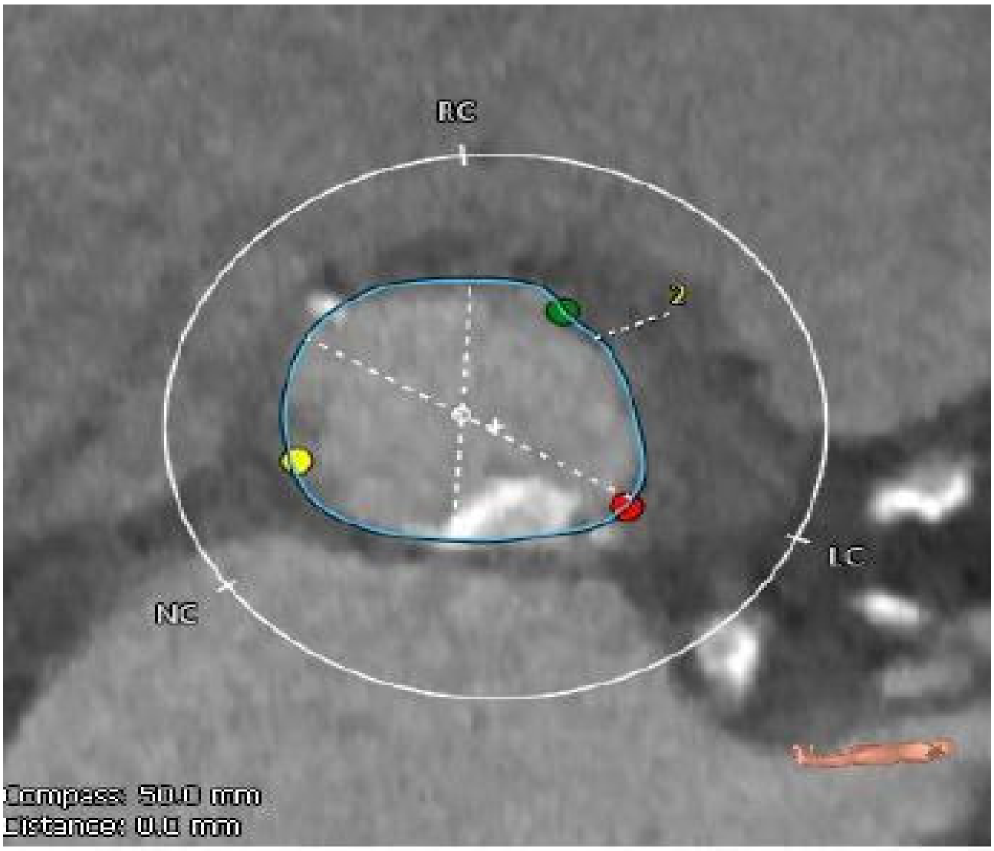

Question 20

Severe calcification(s) of the aortic annulus
- Would this anatomical finding impact your choice of THV? - ○ Yes ○ No
- How suitable would you grade the following THVs?

Sapien 3 (Edwards): -
Corevalve Evolut R (Medtronic): -
Lotus Valve (Boston Scientific): -
Symetis Acurate (Boston Scientific): -
Portico (Abbott): -

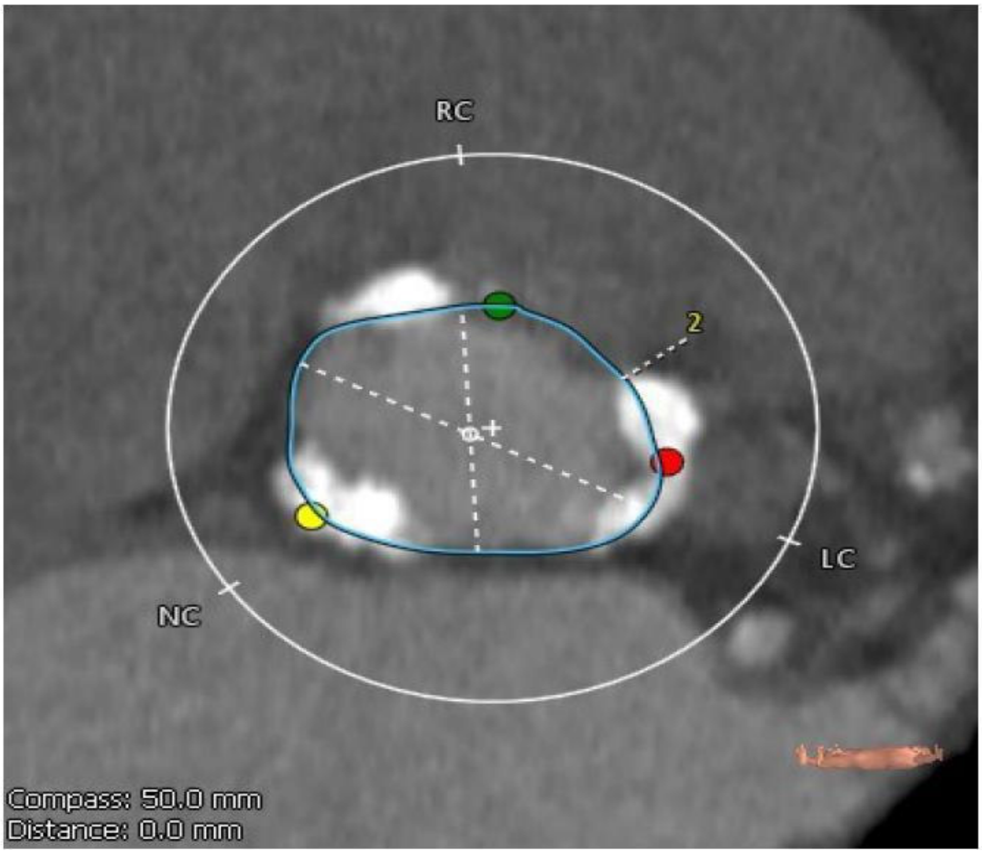

Question 21

Severe calcification(s) > 5 mm protruding into the LVOT

- Would this anatomical finding impact your choice of THV? - ○ Yes ○ No
- How suitable would you grade the following THVs?

Sapien 3 (Edwards): -

Corevalve Evolut R (Medtronic): -

Lotus Valve (Boston Scientific): -

Symetis Acurate (Boston Scientific): -

Portico (Abbott): -

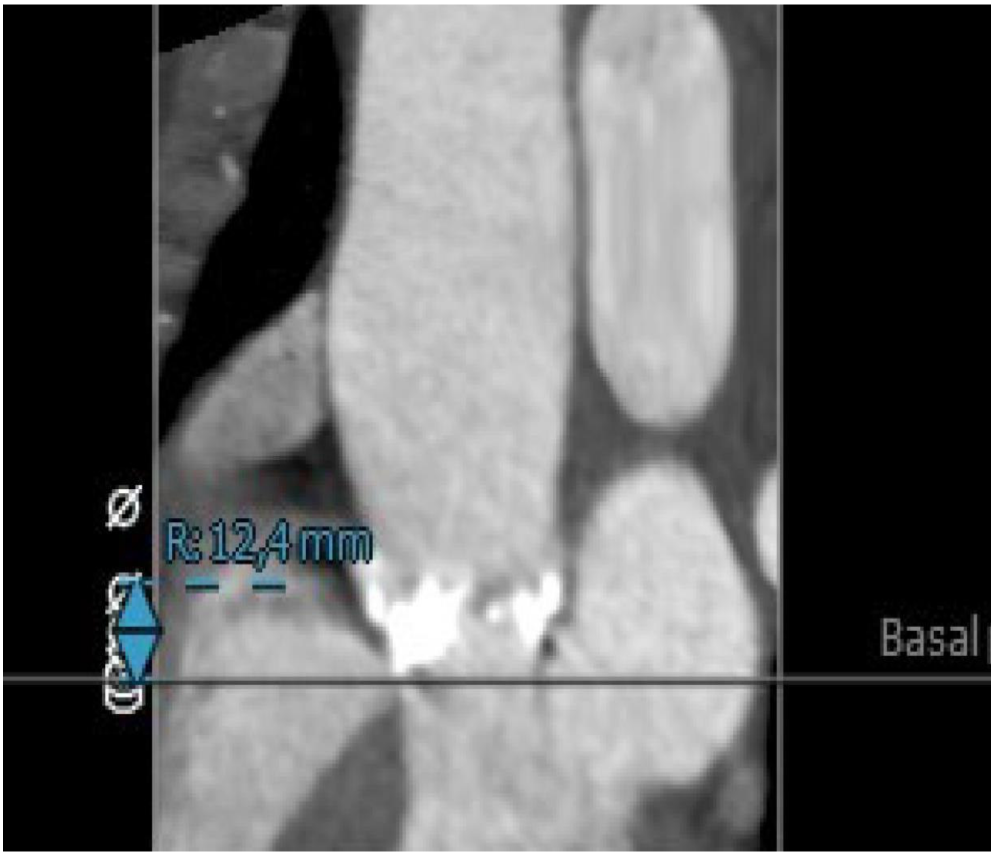

Question 22

Non-calcific severe AS with significant thickening of aortic leaflets
- Would this anatomical finding impact your choice of THV? - ○ Yes ○ No
- How suitable would you grade the following THVs?

Sapien 3 (Edwards): -
Corevalve Evolut R (Medtronic): -
Lotus Valve (Boston Scientific): -
Symetis Acurate (Boston Scientific): -
Portico (Abbott): -

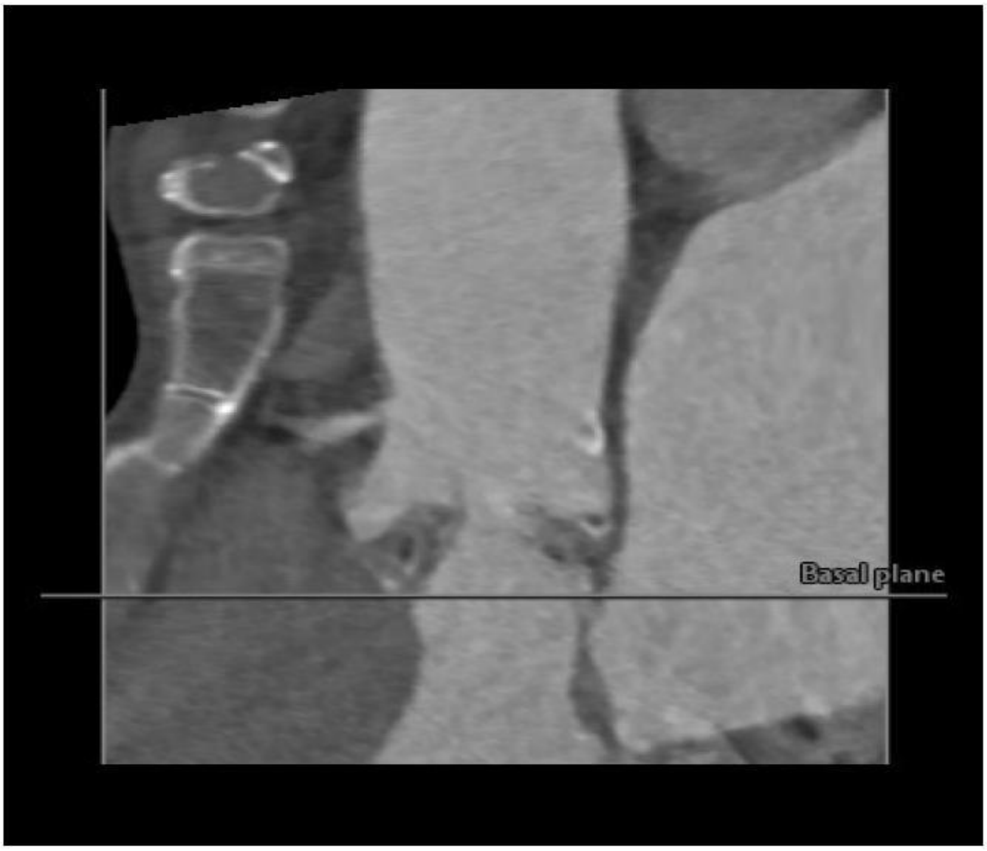

Question 23

Severe, bulky calcifications (> 5mm) at the RCC/LCC native leaflets (coronary cusps)
- Would this anatomical finding impact your choice of THV? - ○ Yes ○ No
- How suitable would you grade the following THVs?

Sapien 3 (Edwards): -
Corevalve Evolut R (Medtronic): -
Lotus Valve (Boston Scientific): -
Symetis Acurate (Boston Scientific): -
Portico (Abbott): -

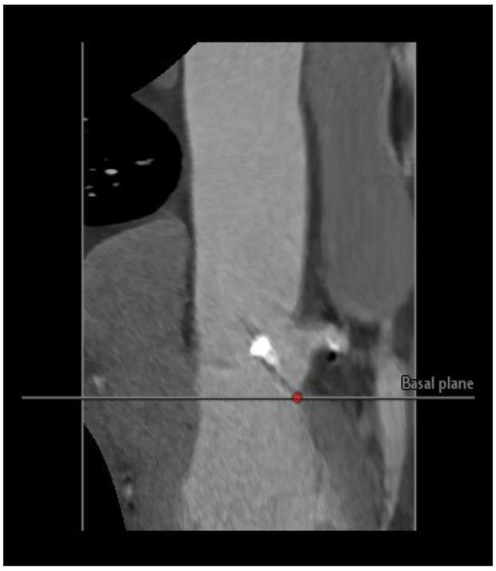

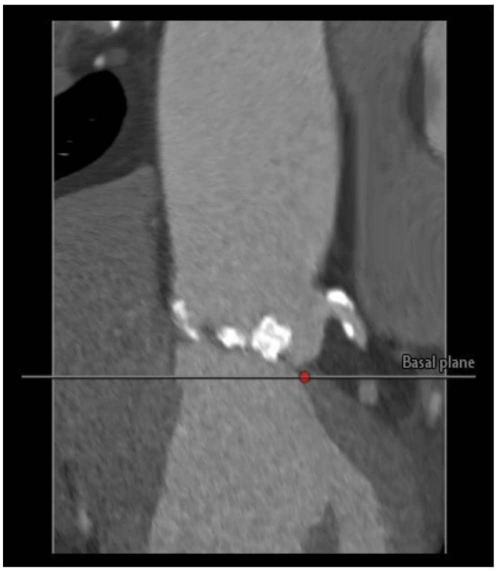

Question 24

Sapien 3 (Edwards): -
Corevalve Evolut R (Medtronic): -
Lotus Valve (Boston Scientific): -
Symetis Acurate (Boston Scientific): -
Portico (Abbott): -

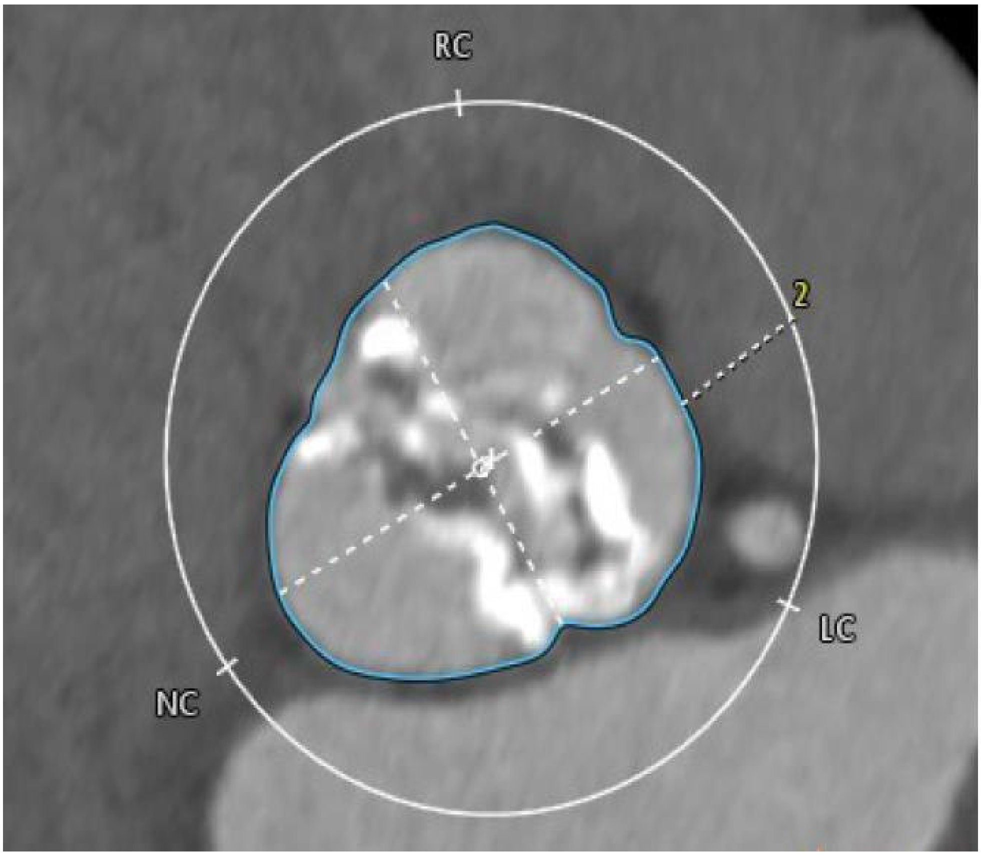

Question 25

Isolated, non-calcific severe native aortic valve regurgitation
- Would this anatomical finding impact your choice of THV? - ○ Yes ○ No
- How suitable would you grade the following THVs?

Sapien 3 (Edwards): -
Corevalve Evolut R (Medtronic): -
Lotus Valve (Boston Scientific): -
Symetis Acurate (Boston Scientific): -
Portico (Abbott): -

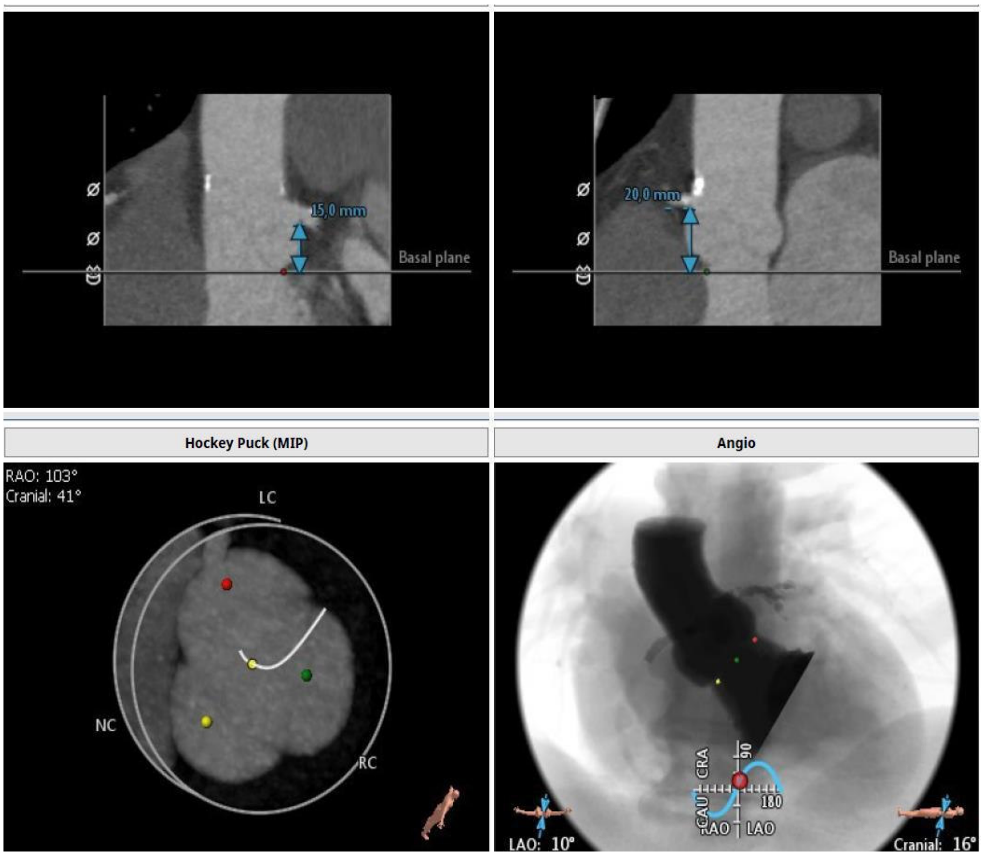

Question 26

TAVI in previous surgical bioprosthesis (valve-in-valve)

- Would this anatomical finding impact your choice of THV? - ○ Yes ○ No
- How suitable would you grade the following THVs?

Sapien 3 (Edwards): -

Corevalve Evolut R (Medtronic): -

Lotus Valve (Boston Scientific): -

Symetis Acurate (Boston Scientific): -

Portico (Abbott): -

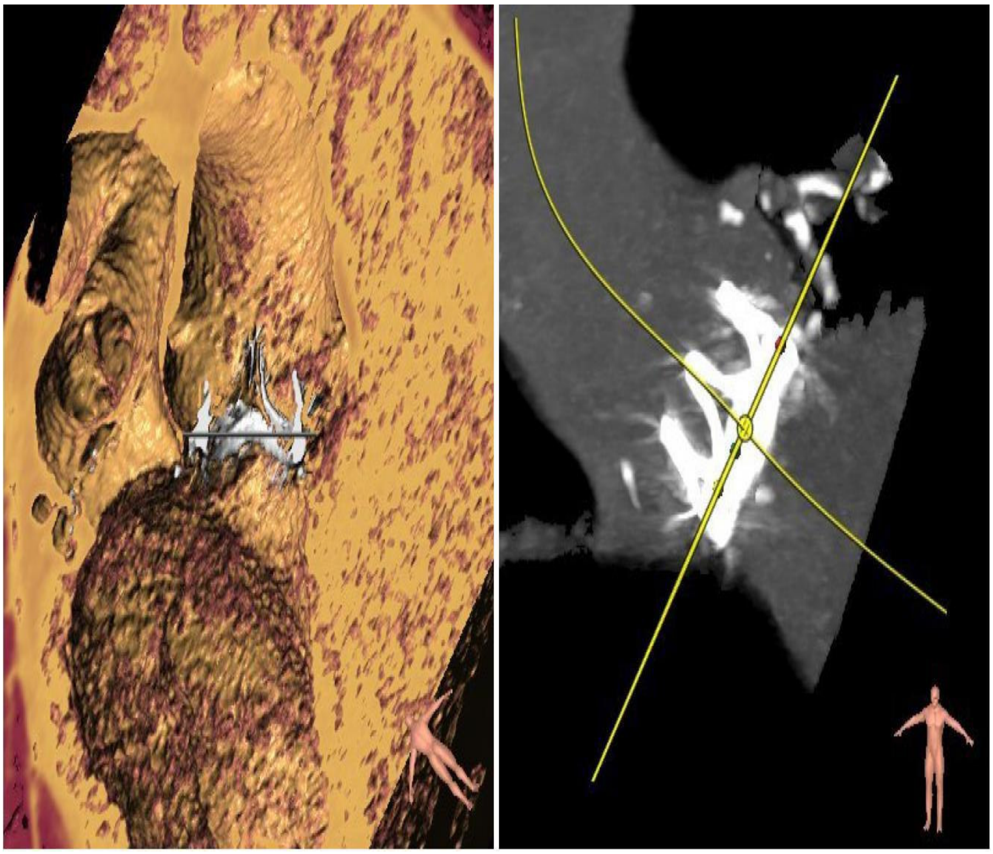

Question 27

Low take-off of coronary arteries (= 7 mm from annular plane)
- Would this anatomical finding impact your choice of THV? - ○ Yes ○ No
- How suitable would you grade the following THVs?

Sapien 3 (Edwards): -
Corevalve Evolut R (Medtronic): -
Lotus Valve (Boston Scientific): -
Symetis Acurate (Boston Scientific): -
Portico (Abbott): -

LM 7 mm

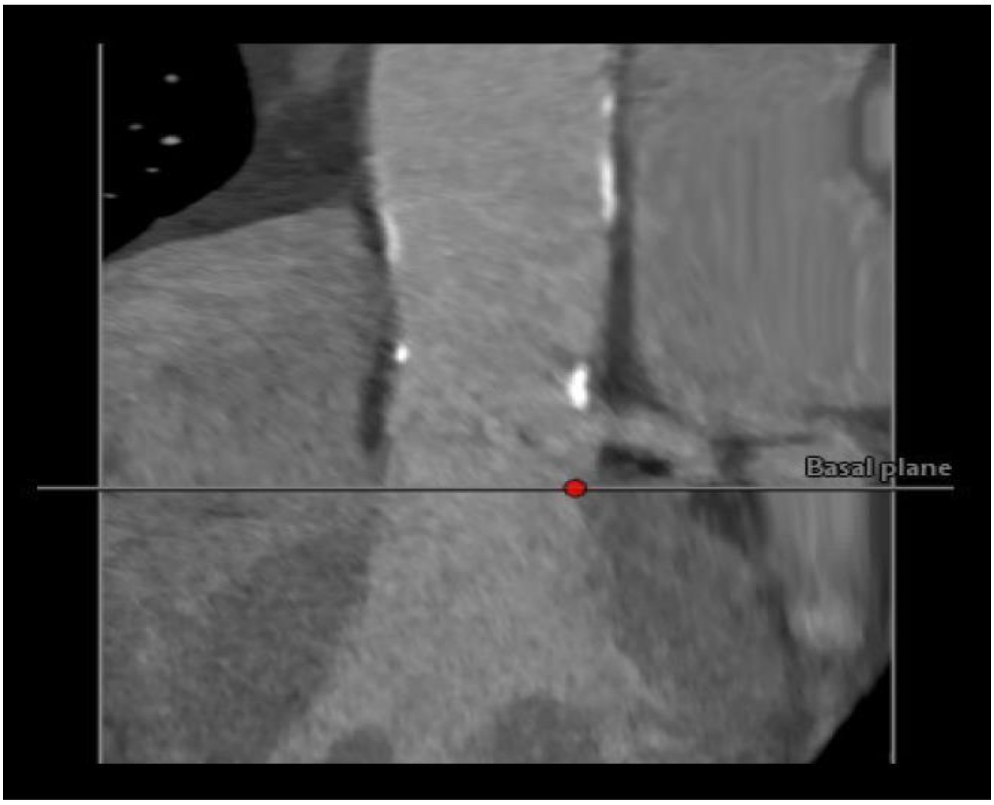

Question 28

Small sinus of Valsalva (mean SoV diameter < 3 mm wider than mean aortic annulus diameter)
- Would this anatomical finding impact your choice of THV? - ○ Yes ○ No
- How suitable would you grade the following THVs?

Sapien 3 (Edwards): -
Corevalve Evolut R (Medtronic): -
Lotus Valve (Boston Scientific): -
Symetis Acurate (Boston Scientific): -
Portico (Abbott): -

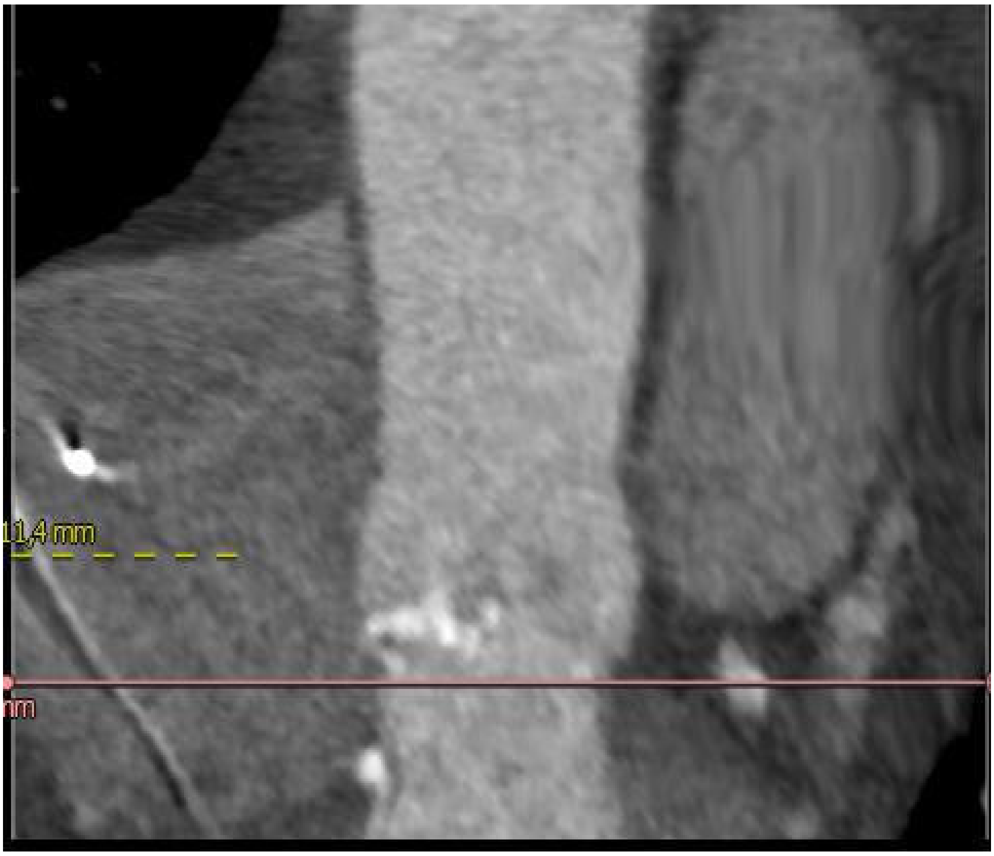

Question 29

Calcified sino-tubular junction with relatively small diameter (< max aortic annulus diameter)
- Would this anatomical finding impact your choice of THV? - ○ Yes ○ No
- How suitable would you grade the following THVs?

Sapien 3 (Edwards): -
Corevalve Evolut R (Medtronic): -
Lotus Valve (Boston Scientific): -
Symetis Acurate (Boston Scientific): -
Portico (Abbott): -

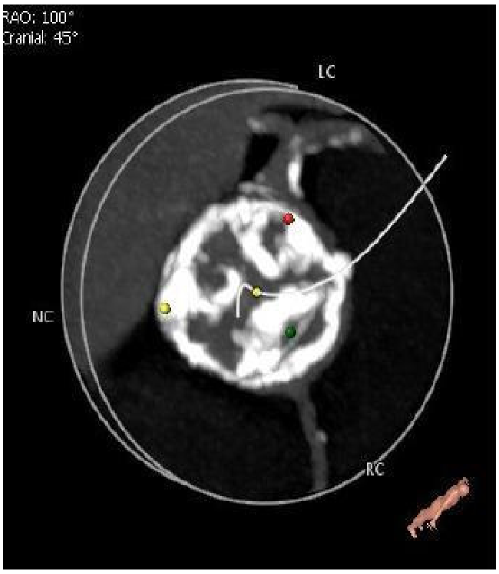

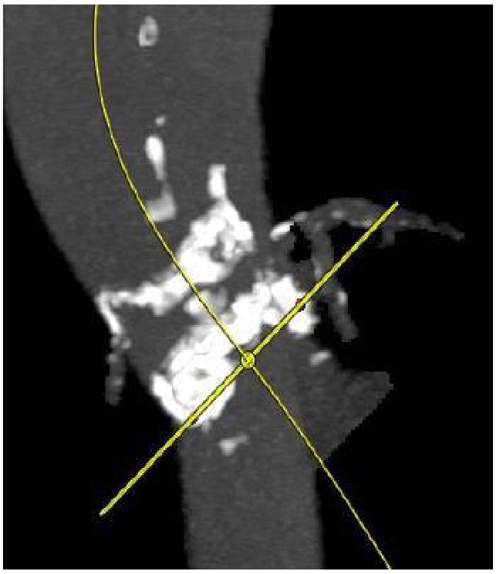

Question 30

Sapien 3 (Edwards): -
Corevalve Evolut R (Medtronic): -
Lotus Valve (Boston Scientific): -
Symetis Acurate (Boston Scientific): -
Portico (Abbott): -

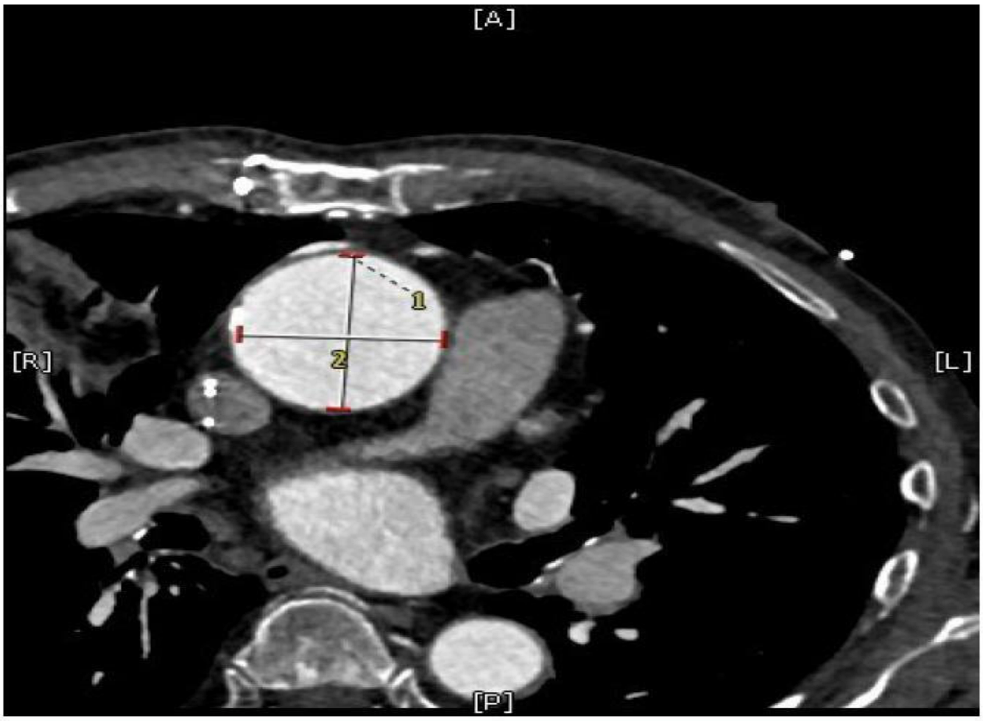

ID	Type	Label	Value
1	Distance		49.7 mm
2	Distance		46.8 mm

Question 31

Moderately reduced LV function (LVEF < 35%)
- Would this knowledge impact your choice of THV? - ○ Yes ○ No
- How suitable would you grade the following THVs?

Sapien 3 (Edwards): -
Corevalve Evolut R (Medtronic): -
Lotus Valve (Boston Scientific): -
Symetis Acurate (Boston Scientific): -
Portico (Abbott): -

Question 32

Severely reduced LV function (LVEF < 20%)
- Would this knowledge impact your choice of THV? - ○ Yes ○ No
- How suitable would you grade the following THVs?

Sapien 3 (Edwards): -
Corevalve Evolut R (Medtronic): -
Lotus Valve (Boston Scientific): -
Symetis Acurate (Boston Scientific): -
Portico (Abbott): -

Question 33

Sapien 3 (Edwards): -
Corevalve Evolut R (Medtronic): -
Lotus Valve (Boston Scientific): -
Symetis Acurate (Boston Scientific): -
Portico (Abbott): -

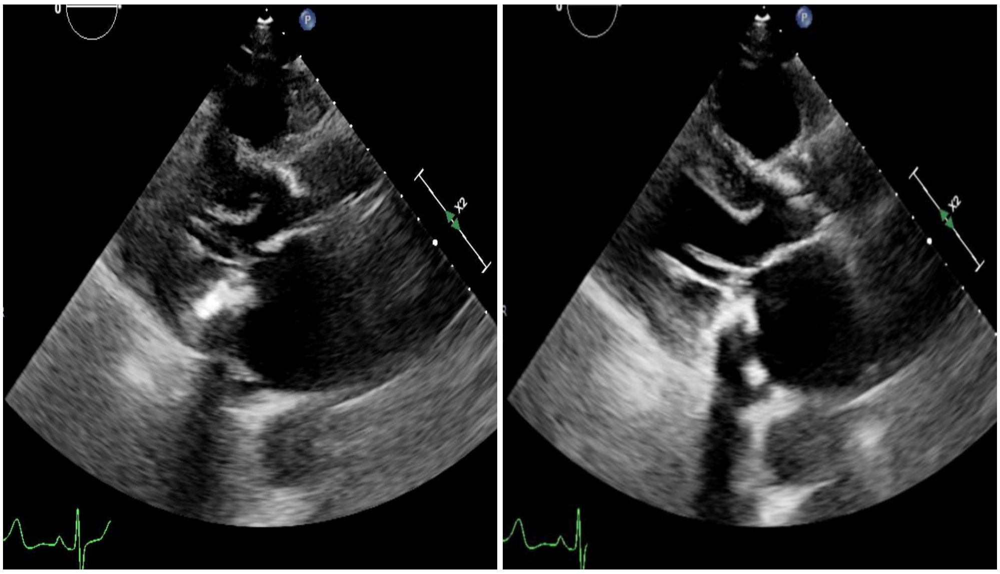

Question 34

Patient with previously known PR-interval > 200ms
- Would this knowledge impact your choice of THV? - ○Yes ○No
- How suitable would you grade the following THVs?

Sapien 3 (Edwards): -
Corevalve Evolut R (Medtronic): -
Lotus Valve (Boston Scientific): -
Symetis Acurate (Boston Scientific): -
Portico (Abbott): -

Question 35

Patient with previously known LBBB
- Would this knowledge impact your choice of THV? - ○Yes ○No
- How suitable would you grade the following THVs?

Sapien 3 (Edwards): -
Corevalve Evolut R (Medtronic): -
Lotus Valve (Boston Scientific): -
Symetis Acurate (Boston Scientific): -
Portico (Abbott): -

Question 36

Patient with previously known RBBB
- Would this knowledge impact your choice of THV? - ○Yes ○No
- How suitable would you grade the following THVs?

Sapien 3 (Edwards): -
Corevalve Evolut R (Medtronic): -
Lotus Valve (Boston Scientific): -
Symetis Acurate (Boston Scientific): -
Portico (Abbott): -

Question 37

Young patient (65-70 years) - with higher risk for redo-TAVI in the future
- Would this knowledge impact your choice of THV? - ○ Yes ○ No
- How suitable would you grade the following THVs?

Sapien 3 (Edwards): -
Corevalve Evolut R (Medtronic): -
Lotus Valve (Boston Scientific): -
Symetis Acurate (Boston Scientific): -
Portico (Abbott): -

Question 38

Patient very likely needing post-TAVI percutaneous coronary intervention (PCI)
- Would this knowledge impact your choice of THV? - ○ Yes ○ No
- How suitable would you grade the following THVs?

Sapien 3 (Edwards): -
Corevalve Evolut R (Medtronic): -
Lotus Valve (Boston Scientific): -
Symetis Acurate (Boston Scientific): -
Portico (Abbott): -

Question 39

How do you evaluate PVL grade right after THV implantation during TAVI procedure? Please indicate the methodology you rely most on with "1", the methodology you rely on second most with "2", etc. Please indicate those methodologies you don't systematically rely on with "0".

○ Transthoracic echocardiography
○ Transoesophageal echocardiography
○ Invasive pressure measurements
○ Contrast aortography

Submit! Reset

Copyright © 2018 mydataservice Survey | Designed by Ska
