# Supplementary material for: WorldwIde SurvEy on Clinical and Anatomical Factors Driving the Choice of Transcatheter Aortic Valve pRostheses
Source: Front Cardiovasc Med. 2020 Mar 20;7:38. doi: 10.3389/fcvm.2020.00038 (PMC7098951; doi:10.3389/fcvm.2020.00038)
Supplement: Supplementary File 2 — Summary of exclusion criteria used in randomized clinical trials/CE mark trials according to different THV devices. [file Table_1.DOCX]

| **Characteristic** | **Clinical relevance** | **Partner I/Partner II** | **SURTAVI** | **Portico-I EU/Portico US** | **REPRISE II** | **DIRECT FLOW US** | **ENGAGER** |
| --- | --- | --- | --- | --- | --- | --- | --- |
| **Small caliber vessel** (Caliper of the femoral/iliac or axillarian/subclavian artery >5.5 and <6.5 mm) | Risk of vascular complications. Impact on the choice betweeen small vs large profile delivery systems. | EXCL: < 7 mm | EXCL: if not compatible with 18 F | Not clearly defined | EXCL:  < 6 mm🡪 23 mm valve  <6.5 mm🡪 27 valve | EXCL:  < 6.5 mm | Not clearly defined |
| **Tortuous vessels** | Risk of vascular complications. Impact on the choice between small vs large and flexible vs stiff delivery systems | Severe tortuosity | Unspecified | Severe tortuosity | Iliofemoral tortuosity | Severe tortuosity | Unspecified |
| **Hyperacute bend of the thoracic aorta** | Risk of aortic dissection/rupture Impact on the choice between flexible vs stiff delivery systems | EXCL: Hyperacute bend of the thoracic aorta | Unspecified | EXCL: Hyperacute bend of the thoracic aorta | EXCL: severe unfolding of the thoracic aorta | Unspecified | Unspecified |
| **Horizontal aorta** (i.e.:angle between aortic annulus and horizontal plane > 70°) | Risk of valve dislodgment. Impact on the choice between self and balloon expandable valves. | Unspecified | EXCL: if angle > 70° | EXCL: if angle > 70° | Unspecified | Unspecified | Unspecified |
| **Left subclavian/ Transcarotid/ Transaortic access** | Need for flexible delivery system. Risk of vascular complication. | NA | NA | NA | NA | NA | NA |
| **Concentric calcifications involving the aorto-iliac bifurcation** | Risk of vascular complication. Impact on the choice between small and flexible vs large and stiff delivery system. | Unspecified | Unspecified | Unspecified | EXCL: “iliofemoral calcifications” | EXCL:” severe calcification of the vascular access” | Unspecified |

| **Characteristic** | **Clinical relevance** | **Partner I/Partner II** | **SURTAVI** | **Portico EU/Portico US** | **REPRISE II** | **DIRECT FLOW US** | **ENGAGER** |
| --- | --- | --- | --- | --- | --- | --- | --- |
| **Large annulus** (Area between 690 mm^3^and 710 mm^3^, perimeter between 90 and 95 mm, max diameter >27 mm) | Impact on potential candidacy for TAVI. Only two commercially availabe prostheses (ES 29 and CV 34) cover annuli with a max diameter above 27 mm | EXCL: > 25 mm | EXCL: > 29 mm | EXCL: > 27 mm | Inclusion: ≤27 mm | EXCL: 26 mm | EXCL: > 27 mm |
| **Small annulus** (Area between 270 mm3 and 300 mm, perimeter between 55 and 60 mm, max diameter between 18 and 19 mm) | Impact on potential candidacy for TAVI. | EXCL:<18 mm | EXCL: <20 mm | EXCL: > 19 mm | Inclusion: ≥19 mm | EXCL: < 19 mm | EXCL: > 18 mm |
| **Circumferential annular calcifications** (Annular calcifications extending for more than 270° of annular perimeter) | Impact on the choice between self expandable and balloon expandable devices. Risk of annular rupture. | Unspecified | Unspecified | Unspecified | Unspecified | Unspecified | Unspecified |
| **Annular Calcifications** (localized at the annular plane or below the annular plane (-5/-7 mm) protruding towards the lumen and with a diameter > 5 mm). | Risk of significant residual PVL after TAVI. | Unspecified | Unspecified | EXCL: extreme eccentricity of calcifications | Unspecified | EXCL: extreme asymmetrical calcification of the native valve | Unspecified |

| **Characteristic** | **Clinical relevance** | **Partner I/Partner II** | **SURTAVI** | **Portico EU/Portico US** | **REPRISE II** | **DIRECT FLOW US** | **ENGAGER** |
| --- | --- | --- | --- | --- | --- | --- | --- |
| **Tandem calcifications**  (protruding annular calcifications (> 5 mm) divided by a perimetral distance of 10-20 mm) | Risk of PVL. Balloon expandable vs self expandable risk of PVL/ low vs high radial force | Unspecified | Unspecified | EXCL: extreme eccentricity of calcifications | Unspecified | EXCL: extreme asymmetrical calcification of the native valve | Unspecified |
| **Arrow headed calcifications**  (arrow headed annular calcifications pointing away from aortic lumen) | Risk of annular rupture during BAV/ valve implant. Impact on the choice between self vs ballon expandable valves | Unspecified | Unspecified | EXCL: extreme eccentricity of calcifications | Unspecified | EXCL: extreme asymmetrical calcification of the native valve | Unspecified |
| **Eccentric aortic annulus** (eccentricity index [1- (min diameter/max diameter)] >0.25) | Risk of PVL. Impact on the choice of Balloon vs self expandable and low vs high radial force valves. | Unspecified | Unspecified | Unspecified | Unspecified | Unspecified | Unspecified |
| **Low implant of coronary arteries**  (LM or RCA take off below 10 mm from annular plane) | Risk of coronary obstruction. Impact on the choice between hour glass shaped vs cilindric valves | Unspecified | Unspecified | Unspecified | Unspecified | Unspecified | Unspecified |
| **Straight sinuses of Valsalva** (mean SOV diameter >2 mm longer than mean aortic annulus diameter; SOV area < 130% aortic annulus area) | Risk of coronary obstruction. Impact on the choice between hour glass shaped vs cilindric valves and depth of implant. | Unspecified | Unspecified | Unspecified | Unspecified | Unspecified | Unspecified |

| **Characteristic** | **Clinical relevance** | **Partner I/Partner II** | **SURTAVI** | **Portico EU/Portico US** | **REPRISE II** | **DIRECT FLOW US** | **ENGAGER** |
| --- | --- | --- | --- | --- | --- | --- | --- |
| **Bulky calcifications of the RCC/LCC native leaflets** (central calcifications of the edge of coronary cusps with a diameter > 5 mm) | Risk of coronary PVL and need for predilatation. Impact on choice between between hour glass shaped vs cilindric valves | Unspecified | Unspecified | Unspecified | Unspecified | Unspecified | Unspecified |
| **Bicuspid aortic valve** | Exclusion criterium in all randomized trials. | EXCL | EXCL | EXCL | EXCL | EXCL | EXCL |
| **Bulky calcified aortic valve leaflets in close proximity to coronary ostia.** | Risk of coronary occlusion. Impact on TAVI candidability and choice between between hour glass shaped vs cilindric valves. | Unspecified | Unspecified | EXCL: Bulky calcified aortic valve leaflets in close proximity to coronary ostia. | Unspecified | Unspecified | Unspecified |
| **Isolated severe aortic valve regurgitation** | AR > moderate exclusion criterium in all randomized trials. Relevant for the choice of valve due to the lack of calcifications and teh presence of larger annuli. | EXCL: AR > 3 | EXCL: AR > 3 | EXCL: AR > 3  EXCL: absence of calcifications | EXCL: AR > 3 | EXCL: AR > 3 | Unspecified |
| **TAVI in biological prosthetic valve** | Exclusion criterium in all randomized trials. | EXCL | EXCL | EXCL | EXCL | EXCL | EXCL |

| **Characteristic** | **Clinical relevance** | **Partner I/Partner II** | **SURTAVI** | **Portico EU/Portico US** | **REPRISE II** | **DIRECT FLOW US** | **ENGAGER** |
| --- | --- | --- | --- | --- | --- | --- | --- |
| **Patients with known ischemic heart disease potentially requiring post implant percutaneous coronary interventions** | Challenging access to coronary ostia after TAVI, particularly with small cells stent design. Impact on the choice between cilindric (short) vs self expandable (long) valves and large vs small cells. | Unspecified | Unspecified | Unspecified | Unspecified | Unspecified | Unspecified |
| **Dilatation of the ascending aorta** (Diameter > 50 mm). | Exclusion criterium of all randomized clinical trials. | PARTNER I  EXCL: > 50 mm  PARTNER II: Unspecified | EXCL : > 43 mm | Portico US: EXCL > 50 mm  Portico EU: EXCL: “Thoracic aneurysm” | EXCL : > 50 mm | EXCL : > 50 mm | EXCL : > 50 mm |
| **Severely hypertrophic LV and short LV** (apex to base distance > 75 mm) | Hypertrofic cardiomiopathy is an exclusion criterium in all randomized clinical trial. Severe hypetrophy is not mentionned. Short and hypetrophic are at potential risk of ventricular perforation. | Unspecified | Unspecified | Unspecified | Unspecified | Unspecified | Unspecified |
| **Poor LV function** (EF between 20 and 30%) | Reduced LV is an exclusion criterium in all randomized trials. Impact on the choice of valve and need for fast pacing | Unspecified | Unspecified | Unspecified | Unspecified | Unspecified | Unspecified |

| **Characteristic** | **Clinical relevance** | **Partner I/Partner II** | **SURTAVI** | **Portico EU/Portico US** | **REPRISE II** | **DIRECT FLOW US** | **ENGAGER** |
| --- | --- | --- | --- | --- | --- | --- | --- |
| **Patients with Right Bundle Branch Block** | Increased risk of complete AV block. Impact on the choice of TAVI | Unspecified | Unspecified | Unspecified | Unspecified | Unspecified | Unspecified |
